# Outcome-based education and student learning in probability and statistics: the mediating roles of engagement and self-efficacy in a new liberal arts context

**DOI:** 10.3389/fpsyg.2026.1817477

**Published:** 2026-05-05

**Authors:** Xuanze Zhao, Hongcheng Ding, Xiaohan Wu, Toong Hai Sam, Joana Jaya

**Affiliations:** 1 School of Information and Artificial Intelligence, Zhejiang University of Finance and Economics Dongfang College, Haining, Zhejiang, China; 2 Faculty of Business, Communication and Law, INTI International University, Nilai, Negeri Sembilan, Malaysia; 3 Faculty of Data Science and Information Technology, INTI International University, Nilai, Negeri Sembilan, Malaysia; 4 School of Accounting, Zhejiang Gongshang University Hangzhou College of Commerce, No.66 Huancheng Road, Hangzhou, Zhejiang, China

**Keywords:** academic self-efficacy, learning outcomes, outcome-based education, probability and statistics education, quasi-experimental design, student engagement, quality education

## Abstract

In the context of pursuing quality education empirical evidence for Outcome-Based Education (OBE) in foundational mathematics is limited, particularly regarding the psychological mechanisms through which it affects learning. This gap is acute within China's New Liberal Arts initiative, a national higher-education reform integrating quantitative reasoning with humanistic and interdisciplinary competencies. This study investigated the association between OBE and three learning outcomes, namely knowledge mastery (KM), statistical application ability (SAA), and cross-disciplinary thinking (CDT), with student engagement (ENG) and self-efficacy (SE) as mediators. A quasi-experimental design was conducted with 321 undergraduates (OBE: *n* = 148; traditional: *n* = 173) at a private university in Eastern China. Analyses included ANCOVA and hierarchical linear modeling for objective examination scores, and partial least-squares structural equation modeling (PLS-SEM, 5,000 bootstraps) for psychological mechanisms. After adjusting for prior mathematics achievement, the OBE group scored significantly higher than the traditional group on the final examination (adjusted mean difference = 6.89, *p* < 0.001), with the advantage confirmed by propensity-score matching (*d* = 0.36, *p* = 0.005). OBE was positively associated with ENG (β = 0.265, *p* < 0.001) and SE (β = 0.276, *p* < 0.001). ENG was identified as a significant statistical mediator in the OBE–KM and OBE–SAA pathways (VAF = 45.7% and 49.5%, respectively), while SE served as a statistical mediator in the OBE–CDT pathway (VAF = 74.0%). Sensitivity analysis supported result robustness (E-value = 3.27). The findings are consistent with a dual-pathway model in which OBE is associated with student learning through a behavioral pathway via ENG to KM and SAA, and a motivational pathway via SE to CDT. A behavioral pathway via ENG to KM and SAA, and a motivational pathway via SE to CDT. Given the quasi-experimental, cross-sectional design, findings should be interpreted as conditional associations pending longitudinal replication.

## 1 Introduction

### 1.1 Background and significance

The reform of mathematics education within the New Liberal Arts represents a critical juncture in higher education, particularly in China, where the integration of quantitative reasoning with humanistic and social scientific perspectives has become increasingly emphasized (Huang, 2024; Clark, 2025). Probability and statistics courses, traditionally taught through lecture-based methods focused on deriving formulas and abstract theorems, face mounting pressure to demonstrate relevance to students' diverse disciplinary backgrounds and future career trajectories (Dinov et al., 2008; Batanero and Álvarez-Arroyo, 2024).

Outcome-Based Education (OBE), first conceptualized by Spady (1994), offers a pedagogical framework that prioritizes clearly defined learning outcomes and aligns curriculum design, instructional activities, and assessment methods accordingly (Farand et al., 2024; Syeed et al., 2022; Kaliannan and Chandran, 2012). This learner-centered approach emphasizes what students can demonstrate upon completion rather than what content instructors have covered. Despite the widespread adoption of OBE in professional education and engineering disciplines, empirical research that examines the implementation of OBE in foundational mathematics courses, particularly in interdisciplinary contexts, remains limited (Harden, 2007; Rao, 2020).

The New Liberal Arts initiative in China calls for breaking down disciplinary silos and cultivating students' ability to apply mathematical thinking across domains (Becker, 2022; Logan and Curry, 2015). This creates a unique context for investigating whether OBE principles can effectively bridge the gap between abstract statistical concepts and practical interdisciplinary applications(Seifert et al., 2008). Furthermore, understanding the psychological and behavioral mechanisms through which OBE influences learning outcomes is essential for optimizing course design and informing broader educational policy.

### 1.2 Research gap

While existing literature has documented positive associations between OBE implementation and student performance in various disciplines, several critical gaps remain:

First, **limited evidence in mathematics education contexts**: Most OBE research focuses on professional programs (e.g., engineering, nursing, business) where learning outcomes are more readily operationalized through practical competencies. The application of OBE to abstract mathematical subjects like probability theory remains underexplored (Chen et al., 2025).

Second, **insufficient understanding of mediating mechanisms**: Existing studies often treat OBE as a black box, measuring only input (teaching method) and output (performance) without examining the psychological and behavioral processes that explain how and why OBE works. The roles of student engagement and self-efficacy as potential mediators have not been systematically investigated in probability and statistics education (Dhandi, 2025).

Third, **lack of differentiated outcome measures**: Previous research typically employs undifferentiated achievement measures (e.g., final grades) without distinguishing among distinct learning outcome dimensions such as foundational knowledge mastery, practical application ability, and higher-order cross-disciplinary thinking (Pinilla et al., 2021).

Fourth, **contextual gap in New Liberal Arts framework**: To our knowledge, empirical studies examining associations between OBE-oriented instruction and student-reported learning processes within the New Liberal Arts context remain limited (Qin, 2023).

### 1.3 Theoretical contributions of this study

This study makes three novel theoretical contributions that advance beyond the current literature. First, it proposes and tests a **dual-pathway mediation model** that differentiates between behavioral (engagement-mediated) and motivational (self-efficacy-mediated) mechanisms through which OBE influences distinct learning outcome dimensions. This represents a significant advance over prior studies that treated OBE as a “black box” or examined only single mediators (Chen et al., 2025; Jordan et al., 2021). Second, it extends OBE research to **foundational mathematics education within the New Liberal Arts context**, a setting that differs fundamentally from the engineering and professional education contexts dominating the existing literature in terms of student population, disciplinary culture, and pedagogical challenges. Third, it adopts a **dual-outcome analytic strategy** that integrates objective academic performance (via ANCOVA, HLM, and PSM) with self-reported psychological mechanisms (via PLS-SEM), providing complementary evidence from both administrative records and student perceptions within a single study.

### 1.4 Research objectives and questions

This study addresses these gaps through the following objectives:

1. To compare the effectiveness of OBE-reformed and traditional teaching approaches in probability and statistics courses using both objective academic performance (standardized final examination scores) and multi-dimensional self-reported learning outcomes.
2. To investigate the mediating roles of student engagement and self-efficacy in the relationship between course modality and learning outcomes.
3. To examine whether OBE principles can effectively promote not only technical competency but also cross-disciplinary thinking capabilities aligned with New Liberal Arts goals.

Specifically, this study addresses three research questions:

**RQ1**:How does OBE course modality influence student engagement (ENG) and self-efficacy (SE) compared to traditional teaching methods?

**RQ2**:What are the direct and indirect effects of OBE course modality on knowledge mastery (KM), statistical application ability (SAA), and cross-disciplinary thinking (CDT)?

**RQ3**: Do engagement (ENG) and self-efficacy (SE) serve as significant mediators in the relationships between OBE course modality and various learning outcomes (KM, SAA, CDT)?

Importantly, this study adopts a dual-outcome analytic strategy: objective academic performance is treated as an external validation of instructional effectiveness, while structural equation modeling is employed to examine the psychological mechanisms underlying observed differences. This integrated design allows for both performance validation and mechanism explanation.

### 1.5 Paper structure

The remainder of this paper is organized as follows: Section 2 reviews relevant theoretical frameworks and empirical literature, leading to the development of our research hypotheses and conceptual model. Section 3 describes the research design, including course reform implementation, data collection procedures, measurement instruments, and analytical approach. Section 4 reports results in two analytic strands: (a) objective academic performance outcomes analyzed via ANCOVA, HLM, and propensity score matching; and (b) PLS-SEM results including measurement model validation and structural model testing. Section 5 discusses the findings in relation to existing literature, explores theoretical and practical implications, addresses methodological robustness and generalizability, and acknowledges remaining limitations. Section 6 concludes with key takeaways and recommendations for mathematics education reform in the New Liberal Arts context.

## 2 Literature review and hypothesis development

### 2.1 Theoretical foundations

#### 2.1.1 Outcome-Based Education (OBE) Theory

Outcome-Based Education, as articulated by Spady (1994), represents a paradigm shift from input-focused to output-focused educational design. The core principle of OBE is that educational structures and curricula should be organized around clearly defined outcomes that students are expected to achieve upon completion. Biggs (2003); Wang et al. (2013) further developed this concept through their theory of constructive alignment, which emphasizes the coherent integration of intended learning outcomes, teaching/learning activities, and assessment tasks.

OBE differs fundamentally from traditional teaching models in several key aspects (Shaheen, 2019; Zamir et al., 2022): (1) it begins with the end in mind by defining what students should be able to do; (2) it emphasizes demonstration of learning through authentic assessment rather than passive knowledge reception; (3) it provides multiple opportunities for students to achieve outcomes through iterative feedback and improvement; and (4) it holds instructors accountable for facilitating student success rather than merely delivering content (Jaya et al., 2025).

#### 2.1.2 New liberal arts framework

The New Liberal Arts movement in China, initiated in 2020, seeks to modernize humanities and social sciences education by integrating emerging technologies, quantitative methods, and interdisciplinary perspectives (Gao, 2021). Unlike traditional liberal arts education that maintains strict disciplinary boundaries, New Liberal Arts emphasizes the cultivation of composite talents who can apply diverse knowledge systems to complex real-world problems (Cheng, 2022; Jing and Tan, 2024).

For probability and statistics courses serving liberal arts students, this framework necessitates several pedagogical adaptations: connecting abstract mathematical concepts to social, economic, and cultural phenomena; developing data literacy skills relevant to students' diverse majors; fostering critical thinking about statistical claims in media and research; and cultivating the ability to translate between mathematical models and domain-specific contexts (Li and Li, 2024; Jing and Tan, 2024).

The alignment between OBE principles and New Liberal Arts goals is conceptually strong, as both emphasize practical competency development, interdisciplinary integration, and learner-centered pedagogy. However, empirical validation of this theoretical alignment remains limited.

#### 2.1.3 Constructivist learning theory

Constructivist learning theory (Efgivia et al., 2021), rooted in the work of Piaget and Vygotsky, posits that learners actively construct knowledge through interaction with their environment rather than passively receiving information. Srikan et al. (2021); Yang et al. (2025) applied constructivist principles to higher education through the concept of constructive alignment, arguing that effective learning occurs when teaching methods and assessment align with intended learning outcomes in ways that support active knowledge construction.

This theoretical perspective is particularly relevant to OBE implementation, as outcome-focused design naturally directs attention to the cognitive processes through which students build understanding (Almulla, 2023). In probability and statistics education, constructivist approaches might include using cognitive conflict to challenge intuitive misconceptions about randomness, scaffolding complex problem-solving through structured inquiry activities, facilitating peer discussion to externalize and refine statistical reasoning, and connecting new concepts to students' prior knowledge from their disciplinary backgrounds (Lee et al., 2025; Bigdeli et al., 2023).

### 2.2 Empirical literature

#### 2.2.1 OBE effectiveness in higher education

A growing body of research has examined OBE implementation across various educational contexts, yielding inconsistent findings that vary by discipline and outcome measure. In engineering education, Jordan et al. (2021) found that OBE-based courses significantly improved students' problem-solving skills and professional competencies compared to traditional lecture-based instruction. Similarly, in medical education, meta-analyses have demonstrated positive effects of outcome-based curricula on clinical reasoning and performance (Li et al., 2021). However, Dinh and Nguyen (2023) reported that OBE implementation in Vietnamese higher education produced only marginal improvements in student satisfaction, with gains concentrated among high-achieving students, raising concerns about equity.

In mathematics education, findings are notably more mixed and sometimes contradictory. While Xu et al. (2024) reported improved conceptual understanding following OBE reforms in calculus courses, a systematic review by Mouta et al. (2025) identified significant implementation challenges including instructor resistance, student adjustment difficulties, and concerns about depth of theoretical coverage. Critically, Chen et al. (2025) found that OBE effects on mathematical problem-solving were fully mediated by student motivation, suggesting that the “black box” between OBE and learning outcomes contains psychological processes that most studies fail to examine. This inconsistency highlights two key gaps: (1) most studies treat OBE as a uniform intervention without examining *how* and *why* it works, and (2) disciplines with strong applied orientations (engineering, nursing) may show larger OBE effects because outcome operationalization is more straightforward than in abstract mathematical subjects.

Our proposed model addresses these inconsistencies by explicitly specifying the mediating mechanisms (engagement and self-efficacy) through which OBE influences learning outcomes, rather than treating OBE as a monolithic input variable. Unlike prior models that examined only direct OBE—performance links (Jordan et al., 2021) or single mediators (Chen et al., 2025), our dual-pathway framework differentiates between behavioral (engagement) and motivational (self-efficacy) mechanisms, providing a more nuanced theoretical account of OBE's differential effects on distinct outcome dimensions.

#### 2.2.2 Student engagement as a learning mechanism

Student engagement, conceptualized by Farikah et al. (2023) as comprising behavioral, emotional, and cognitive dimensions, has been consistently linked to positive learning outcomes across disciplines. Engaged students invest effort in learning activities, persist through challenges, experience positive emotions about learning, and employ deep cognitive strategies.

In statistics education research, engagement has been specifically recognized as a critical factor in overcoming student anxiety and fostering conceptual understanding (Mengesha et al., 2024). Active learning approaches, which promote engagement through methods such as data-based projects, collaborative problem-solving, and interactive simulations, have shown to be more effective than passive lecture methods in developing statistical reasoning (Afroze and Shafi, 2024).

The relationship between OBE and engagement is theoretically grounded in self-determination theory (Taylor and King, 2023), which suggests that learning environments supporting autonomy, competence, and relatedness foster intrinsic motivation and engagement. OBE's emphasis on clear learning targets, authentic tasks, and formative feedback aligns with these psychological needs.

However, the literature reveals important inconsistencies regarding the engagement—performance link in statistics education. While Afroze and Shafi (2024) reported strong positive effects of active learning on statistical reasoning, Kaufmann et al. (2022) found that engagement alone was insufficient to overcome statistics anxiety, suggesting that motivational beliefs (particularly self-efficacy) may play an independent role. Moreover, Mengesha et al. (2024) cautioned that engagement effects may be confounded with prior interest and ability, a concern that is particularly relevant in non-randomized studies. These mixed findings underscore the need to model engagement alongside complementary psychological mechanisms rather than treating it as a standalone predictor.

#### 2.2.3 Self-efficacy in mathematics learning

Self-efficacy, defined by Bandura (2023) as one's belief in their capability to execute specific tasks successfully, has been extensively studied in mathematics education. Research consistently demonstrates that mathematics self-efficacy predicts performance, persistence, and course selection even after controlling for prior achievement (McCaughey et al., 2022; Azzarello et al., 2025).

In probability and statistics specifically, self-efficacy is particularly relevant due to the widespread phenomenon of statistics anxiety and negative attitudes (Kaufmann et al., 2022). Students with higher self-efficacy are more likely to view challenging problems as opportunities for growth rather than threats, to persist through difficult concepts, to seek help when needed, and to transfer statistical knowledge to new contexts.

OBE may enhance self-efficacy through multiple mechanisms including mastery experiences provided by scaffolded, achievable learning outcomes; vicarious experiences from peer modeling in collaborative activities; verbal persuasion through encouraging, specific feedback; and reduced anxiety through transparent expectations and multiple success opportunities.

Despite the extensive literature on self-efficacy in mathematics, two critical limitations persist. First, most studies employ cross-sectional designs that cannot disentangle whether self-efficacy drives performance or whether prior performance shapes self-efficacy (Talsma et al., 2018). Second, the relationship between self-efficacy and higher-order thinking (as opposed to basic computational performance) remains underexplored, with Azzarello et al. (2025) noting that “the mechanisms linking self-efficacy to integrative and transfer outcomes are largely unspecified in current models.” Our study addresses this gap by hypothesizing a specific self-efficacy pathway to cross-disciplinary thinking, an outcome dimension that requires confidence to venture beyond disciplinary boundaries.

#### 2.2.4 Cross-disciplinary thinking in new liberal arts

Cross-disciplinary thinking, which is the capacity to combine knowledge and methods from diverse disciplines to tackle intricate problems, is a crucial competency within New Liberal Arts education. Kim et al. (2024) pinpointed several features of effective cross-disciplinary teaching. These encompass authentic and complex problems demanding multiple viewpoints, explicit focus on disciplinary presuppositions and approaches, well-structured integration opportunities, and metacognitive contemplation on the interdisciplinary process (Rothinam et al., 2025).

In the context of probability and statistics for liberal arts students, cross-disciplinary thinking involves recognizing statistical patterns in social phenomena, evaluating quantitative evidence in policy debates, applying probabilistic reasoning to ethical dilemmas, and integrating mathematical models with domain-specific theories. Limited research has examined how pedagogical approaches influence the development of such capabilities, representing a significant gap this study addresses (Qiu et al., 2025).

### 2.3 Research hypotheses and conceptual model

#### 2.3.1 Conceptual model and hypotheses development

**Figure 1:**
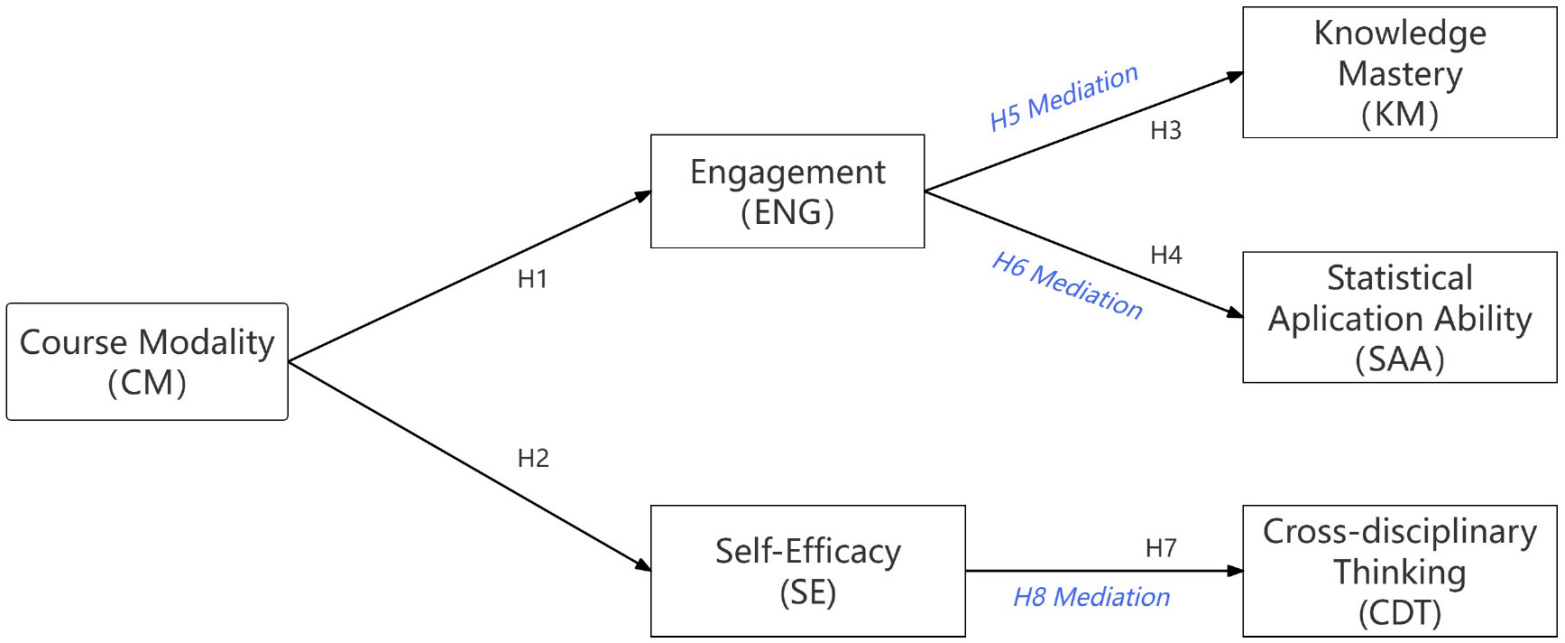
Conceptual model of the primary pathways linking course modality to learning outcomes. Course modality (CM; OBE-reformed vs. traditional teaching) is hypothesized to influence student engagement (ENG) and self-efficacy (SE). Engagement is specified as the primary proximal pathway predicting knowledge mastery (KM) and statistical application ability (SAA), whereas self-efficacy is specified as the primary motivational pathway predicting cross-disciplinary thinking (CDT). Dashed arrows indicate theoretically plausible cross-links (ENG → CDT, SE → KM, SE → SAA) that are not included in the focal model for parsimony but are tested in competing-model analyses (see Table 1 and Section 4).

Based on the theoretical frameworks and empirical evidence reviewed above, we propose a dual-pathway model (Figure 1). In this model, the OBE course modality affects three distinct learning outcomes (namely, knowledge mastery, statistical application ability, and cross-disciplinary thinking) through two mediating mechanisms: student engagement and self-efficacy.

We conceptualize student engagement as an enacted, situation-specific process that reflects students' behavioral and cognitive investment in course activities (e.g., effort expenditure, persistence, and deep processing). As such, engagement is theorized to be more proximally linked to within-domain learning outcomes that depend on sustained practice and cognitive processing in the course, namely knowledge mastery (KM) and statistical application ability (SAA). In contrast, self-efficacy is conceptualized as a higher-order motivational belief and self-regulatory resource that shapes students' willingness to take on challenging tasks, persist in the face of uncertainty, and monitor their own learning. These functions are particularly critical for cross-disciplinary thinking (CDT) in the New Liberal Arts context, which requires learners to venture beyond familiar disciplinary boundaries, tolerate ambiguity, and integrate diverse perspectives.

We acknowledge that self-efficacy may also support KM/SAA through increased persistence, and engagement may contribute to CDT through deeper cognitive involvement. However, to maintain theoretical focus and model parsimony, we specify the above primary pathways. The reasons for this focal model specification are as follows.

First, for theoretical parsimony with clear predictions, the dual-pathway specification generates distinct, falsifiable predictions about which mediator primarily explains which outcome. A fully cross-linked model would conflate the unique explanatory contributions of engagement and self-efficacy (Kline, 2023). Second, considering statistical identifiability, given the modest sample size (*N* = 321) and the cross-sectional measurement of all mediators and outcomes, a fully cross-linked model risks overfitting and producing unstable parameter estimates (Hair et al., 2019). Third, for empirical testability, by specifying the most parsimonious model as the focal analysis, we can formally test whether adding cross-links meaningfully improves model fit, which we report as supplementary competing-model analyses (Table 1).

**Table 1:** Competing structural specifications estimated with standardized composite scores (based on provided CSV data, *N* = 321).

Model	Path	β	SE	95% CI	*R* ^2^
Focal (single-antecedent)	CM → ENG	0.456	0.050	[0.358, 0.554]	0.208
Focal (single-antecedent)	CM → SE	0.455	0.050	[0.357, 0.553]	0.207
Focal (single-antecedent)	ENG → KM	0.860	0.028	[0.805, 0.915]	0.740
Focal (single-antecedent)	ENG → SAA	0.897	0.024	[0.850, 0.944]	0.804
Focal (single-antecedent)	SE → CDT	0.871	0.027	[0.818, 0.924]	0.759
Competing A (cross-links)	ENG → KM	0.610	0.067	[0.479, 0.741]	0.740
Competing A (cross-links)	SE → KM	0.276	0.067	[0.145, 0.407]	0.740
Competing A (cross-links)	ENG → SAA	0.710	0.054	[0.604, 0.816]	0.804
Competing A (cross-links)	SE → SAA	0.204	0.054	[0.098, 0.310]	0.804
Competing A (cross-links)	ENG → CDT	0.754	0.045	[0.666, 0.842]	0.759
Competing A (cross-links)	SE → CDT	0.192	0.045	[0.104, 0.280]	0.759
Competing B (2nd-order PLO)	ENG → PLO	0.691	0.043	[0.607, 0.775]	0.865
Competing B (2nd-order PLO)	SE → PLO	0.214	0.043	[0.130, 0.298]	0.865

The competing-model results (Section 4) confirm that (a) adding cross-links attenuates the focal path coefficients, as expected when shared variance is redistributed, (b) engagement emerges as a stronger general predictor than self-efficacy when cross-links are allowed, and (c) the substantive conclusion that OBE is positively associated with perceived learning outcomes through both behavioral and motivational pathways remains stable across specifications.

It is important to note that the proposed “dual-pathway” structure is intended to distinguish primary (more proximal) mechanisms rather than to imply exclusive effects. We also discuss the potential cross-links as directions for future research.

#### 2.3.2 Direct effects of OBE course modality

**H1:** OBE course modality has a significant positive effect on student engagement (ENG).

OBE (Outcome-Based Education) emphasizes active, student-centered learning activities that are aligned with authentic outcomes. These design features, including problem-based learning, collaborative projects, and interactive discussions, are both theoretically and empirically linked to higher levels of behavioral, emotional, and cognitive engagement than traditional lecture-based instruction. Drawing on self-determination theory, we propose that OBE's clarity regarding learning targets (which supports competence), the provision of student choice in demonstration methods (which supports autonomy), and collaborative activities (which support relatedness) will foster enhanced engagement.

**H2:** OBE course modality has a significant positive effect on self-efficacy (SE).

OBE provides clear learning outcomes, scaffolded learning progressions, and regular formative feedback, all of which are recognized sources of self-efficacy according to (Bandura, 2023). By breaking complex competencies into achievable milestones and offering multiple opportunities to demonstrate mastery, OBE creates mastery experiences, which are the most powerful contributors to building self-efficacy. Additionally, the collaborative learning inherent in OBE implementation offers opportunities for vicarious learning and social persuasion.

**H3:** Engagement (ENG) has a significant positive effect on knowledge mastery (KM).

Engagement captures students' enacted cognitive investment (e.g., deep processing and sustained effort), which directly supports conceptual understanding and durable knowledge acquisition in probability and statistics.

**H4:** Engagement (ENG) has a significant positive effect on statistical application ability (SAA).

Engagement facilitates the transfer of statistical knowledge to real-world contexts. Engaged students seek connections between course concepts and practical applications, aiding persistence and problem-solving in new situations.

**H5:** Engagement (ENG) mediates the relationship between OBE course modality and knowledge mastery (KM).

We hypothesize that OBE enhances knowledge mastery not only through direct instructional features but also indirectly by fostering engagement, which in turn promotes the deep cognitive processing necessary for conceptual understanding. This mediation hypothesis suggests that engagement is a key mechanism through which OBE achieves its effects.

**H6:** Engagement (ENG) mediates the relationship between OBE course modality and statistical application ability (SAA).

Similarly, we expect that OBE promotes statistical application ability partially through increased engagement. While OBE's emphasis on authentic tasks directly supports the development of application skills, the motivational benefits of engagement amplify students' willingness to invest in the challenging work of transferring knowledge to new contexts.

#### 2.3.3 Mediating mechanisms: behavioral pathway (engagement) vs. psychological pathway (self-efficacy)

**H7:** Self-efficacy (SE) has a significant positive effect on cross-disciplinary thinking (CDT).

Cross-disciplinary thinking requires students to venture beyond familiar disciplinary boundaries, tolerate ambiguity, and integrate diverse knowledge systems. These are all cognitively and emotionally demanding activities. Students with higher self-efficacy are more likely to embrace these challenges, viewing them as opportunities for growth rather than threats. Zimmerman (2000) demonstrated that self-efficacy predicts self-regulated learning behaviors, including the metacognitive monitoring essential for recognizing when and how to apply knowledge across contexts.

Although engagement may also foster CDT by prompting deeper involvement in integrative tasks, we argue that self-efficacy is a particularly central resource for CDT because interdisciplinary integration often entails uncertainty, higher perceived risk of failure, and the need for metacognitive monitoring. Learners who believe they can succeed are more likely to initiate and sustain the boundary-crossing efforts required for cross-disciplinary integration.

**H8:** Self-efficacy (SE) mediates the relationship between OBE course modality and cross-disciplinary thinking (CDT).

We propose that OBE cultivates cross-disciplinary thinking not only through explicit integration of interdisciplinary content but also by building the confidence students need to apply statistical reasoning beyond mathematics contexts. This mediation hypothesis suggests that psychological empowerment is as important as instructional design in fostering transfer and interdisciplinary application.

This conceptual model represents a comprehensive framework for understanding how pedagogical innovation influences multiple dimensions of learning through distinct psychological and behavioral mechanisms. The differentiation between engagement-mediated effects on foundational and application outcomes vs. self-efficacy-mediated effects on higher-order cross-disciplinary thinking reflects the multifaceted nature of learning in mathematics education.

## 3 Methodology

### 3.1 Research design and participants

This study employed a quasi-experimental design with administrative assignment at the class-section level. This design allows for adjusted group comparisons but does not involve random assignment. The study was part of an institutional teaching innovation project that integrated Chinese proverbs, idioms, and fables into probability and statistics instruction. The following sections outline the group assignment procedure, the temporal sequence of events, and key characteristics of the participants involved in the study.

#### 3.1.1 Assignment procedure and temporal sequence

A critical methodological feature of this study is the temporal sequence of group assignment relative to baseline measurement. At the beginning of the Autumn 2024 semester, the teaching innovation project team selected class sections to receive OBE-reformed instruction based solely on administrative scheduling constraints (e.g., classroom availability, timetable compatibility across colleges). This selection occurred *before* students' prior mathematics achievement scores (Calculus II, completed in Fall 2023) were compiled and made available to the research team.

Specifically, the assignment timeline was as follows:

1. **Week 0 (Pre-semester):** Class sections were designated as OBE or traditional based on scheduling feasibility and project resource allocation, without access to students' academic records.
2. **Week 1:** Instruction commenced in both conditions.
3. **Week 4:** Fall 2023 final grades (including Calculus II) were officially released and accessed by the research team for baseline comparison.

This temporal separation ensures that the observed baseline difference in prior achievement is a post-hoc discovery rather than a selection criterion, thereby ruling out deliberate ability-based sorting as a confounding mechanism (Shadish, 2002; Steiner et al., 2010).

#### 3.1.2 Instructional consistency

Both the OBE-reformed group and the traditional group were taught by the same instructor, followed the same syllabus, used identical teaching resources (textbooks, Supplementary Materials), and adopted the same assessment scheme and grading weights. Classes from different academic majors were centrally scheduled and taught in parallel, ensuring equivalent instructional time and exposure. Importantly, the same prerequisite course (Calculus II) and the same probability and statistics final examination were administered across groups, ensuring measurement equivalence.

Group membership was coded as a binary variable (reform = 1 for OBE; reform = 0 for traditional). Students' prior academic achievement (Calculus II scores) and course performance (final examination scores) were extracted from institutional records, minimizing self-report bias for objective outcomes.

#### 3.1.3 Sampling frame and sample characteristics

##### 3.1.3.1 Sampling Frame

The study population comprised undergraduate students enrolled in probability and statistics courses at a private university in Eastern China during the 2024–2025 academic year. This university is a comprehensive institution offering programs across various disciplines.

Probability and statistics is a required course for most majors, typically taken in the second or third year. Students majoring in liberal arts and social science constitute the target population for this study, aligning with the New Liberal Arts focus.

##### 3.1.3.2 Sample selection

The study compared two groups of students enrolled in Autumn 2024 course sections: an OBE reform group comprising 148 students in sections restructured according to outcome-based education principles, and a traditional teaching group comprising 173 students enrolled in conventionally designed sections. The final analytic sample consisted of 321 undergraduate students (148 in the OBE group and 173 in the traditional group). Power analysis indicated sufficient statistical power (>0.95) to detect small-to-medium effects (Cohen's *f*^2^ ≥ 0.02) at α = 0.05.

### 3.2 Baseline equivalence and covariate selection

To address potential selection bias inherent in quasi-experimental designs, we implemented several methodological safeguards in line with recommendations from the educational research methodology literature (Campbell and Stanley, 2015; Steiner et al., 2010; Msaouel et al., 2023).

#### 3.2.1 Nature of the baseline difference

Baseline comparison revealed that the OBE group had significantly higher prior mathematics achievement than the traditional group (see Table 2).

**Table 2:** Baseline comparison: prior mathematics achievement (calculus II).

Group	*n*	M	SD	t	*p*	Cohen's d
Traditional	173	63.22	18.76	-5.02	<0.001	-0.56
OBE reform	148	73.24	16.79			

95% CI for mean difference: [-13.95, -6.09].

This interpretation is consistent with the methodological literature on quasi-experiments, which recognizes that even random or quasi-random assignment can produce baseline imbalances in finite samples (Rubin, 2008; Austin, 2011). When assignment is demonstrably independent of the outcome-relevant covariate (as established by the temporal sequence in this study), statistical adjustment via ANCOVA provides unbiased estimates of treatment effects under standard assumptions (Jiang et al., 2019).

#### 3.2.2 Covariate adjustment strategy

Students' scores on Calculus II (the prerequisite course completed in the preceding semester) served as the primary covariate for the following reasons:

1. It directly assesses mathematical competency relevant to probability and statistics learning;
2. It was measured before the intervention, precluding reverse causality;
3. It represents an objective, standardized assessment comparable across groups (Kane et al., 2020).

Analysis of Covariance (ANCOVA) was used to adjust for baseline differences when comparing final examination performance. Given that the assignment was independent of prior achievement, ANCOVA yields an unbiased estimate of the average treatment effect under the assumption of no unmeasured confounders correlated with both group assignment and outcomes (Tabachnick et al., 2007; Murnane and Willett, 2010).

##### 3.2.2.1 Scope and limitations of the covariate adjustment

While Calculus II scores represent the most direct and relevant measure of prior mathematical competency, we acknowledge that they do not capture all potential confounders. Specifically, several sources of residual confounding remain:

**Class-level differences**: Although both groups were taught by the same instructor using identical materials and assessments, intact class sections may differ in peer composition, group dynamics, and emergent learning climate. We address this concern through hierarchical linear modeling (HLM), which accounts for class-level clustering (see Section 4.2.6).

**Major-level differences**: Students from different academic majors may bring varying levels of quantitative preparation, motivation, and disciplinary orientation that are not fully captured by a single Calculus II score. While demographic comparisons (Table 3) showed no significant differences in major distribution between groups (χ^2^ = 2.15, *p* = 0.54), unmeasured major-specific effects (e.g., departmental expectations, peer norms regarding mathematics) cannot be entirely excluded.

**Table 3:** Demographic characteristics by group.

Characteristic	OBE Group (*n* = 148)	Traditional Group (*n* = 173)	Test Statistic
Gender, n (%)			χ^2^ = 0.32
- Female	89 (60.1%)	105 (60.7%)	*p* = 0.57
- Male	59 (39.9%)	68 (39.3%)	
Major Category, n (%)			χ^2^ = 2.15
- Humanities	47 (31.8%)	52 (30.1%)	*p* = 0.54
- Social Sciences	63 (42.6%)	75 (43.4%)	
- Other Liberal Arts	38 (25.7%)	46 (26.6%)	
Academic Year, n (%)			χ^2^ = 1.87
- Sophomore	92 (62.2%)	108 (62.4%)	*p* = 0.40
- Junior	56 (37.8%)	65 (37.6%)	

**Peer environment effects**: The collaborative nature of OBE instruction means that individual outcomes may be influenced by the collective characteristics of classmates. While the same-instructor, same-semester design controls for many contextual factors, we cannot rule out the possibility that the OBE sections benefited from more favorable peer environments.

These residual threats to internal validity are acknowledged as limitations (Section 5.4.3) and motivate our use of multiple analytical approaches (ANCOVA, HLM, PSM) to triangulate findings and assess robustness to different assumptions about the confounding structure (Steiner et al., 2010; Shadish, 2002).

#### 3.2.3 Propensity score matching as robustness check

As a complementary robustness check, we implemented 1:1 nearest-neighbor propensity score matching based on prior achievement scores (caliper = 5 points). This method creates a matched sample with improved covariate balance, providing a more stringent counterfactual comparison (Rosenbaum and Rubin, 1983; Austin, 2011). Consistency of findings across ANCOVA and PSM analyses strengthens confidence in the causal interpretation.

### 3.3 Course reform implementation

The OBE-reformed course incorporated several key pedagogical innovations aligned with outcome-based principles and New Liberal Arts goals:

#### 3.3.1 Learning outcome definition

Rather than organizing content around traditional mathematical topics, the OBE course articulated six learning outcomes that collectively underscored the demonstration of competency in authentic contexts rather than the recitation of abstract theorems.

Specifically, the course aimed to cultivate students' ability to apply probability concepts for modeling uncertainty in real-world phenomena across disciplines, interpret and critically evaluate statistical information in media, research, and policy settings, select and implement appropriate statistical methods for analyzing data from their respective disciplinary fields, communicate statistical reasoning and results effectively to diverse audiences, recognize ethical considerations in data collection, analysis, and interpretation, and integrate probabilistic thinking with humanistic perspectives on risk, decision-making, and social justice.

These outcomes emphasized demonstrating competency in authentic contexts rather than reciting abstract theorems.

#### 3.3.2 Pedagogical strategies

To align with OBE principles and New Liberal Arts goals, the reformed course integrated multiple student-centered pedagogical strategies that connected abstract statistical concepts to real-world contexts and cultural backgrounds.

##### 3.3.2.1 Cultural integration with chinese proverbs and idioms

This strategy linked probability concepts to cultural wisdom through structured interpretation and application steps: (1) Concept Mapping: Identify core probability concepts (e.g., conditional probability, compound events) and match them with culturally resonant proverbs/idioms; (2) Contextual Explanation: Introduce the cultural background of the proverb (e.g., “a blessing in disguise” for conditional probability) and its logical connection to the mathematical concept; (3) Example Derivation: Use the proverb scenario to derive mathematical formulas (e.g., Bayesian updating from “Sai Weng's Lost Horse”); (4) Practice Extension: Design similar cultural scenarios for students to apply the concept independently. Key applications included “Three cobblers with their wits combined equal Zhuge Liang” for compound events and “Strike first to gain advantage, delay to suffer disadvantage” for event independence (see Appendix Table 15 for full mapping).

##### 3.3.2.2 Contemporary case-based learning

Real-world cases were embedded following a four-stage implementation framework: (1) Case Selection: Curate cases aligned with students' majors (e.g., epidemic modeling for public health, social media data analysis for media studies); (2) Problem Framing: Convert cases into statistical questions (e.g., “What is the probability of epidemic spread under different intervention measures?”); (3) Guided Inquiry: Provide data sources and analytical tools to guide students through hypothesis testing, model building, and result interpretation; (4) Reflection Discussion: Connect case results to disciplinary practices and societal impacts. Core cases included COVID-19 transmission probability models, legal-reasoning probability analysis, and social equity assessment using statistical data.

##### 3.3.2.3 Ideological and political education integration

Statistical concepts were linked to societal values through targeted design: (1) Value Alignment: Identify statistical principles related to national development (e.g., national statistical achievements, public health policy); (2) Content Integration: Embed value-related questions in instruction (e.g., “How does probability theory support equitable resource allocation?”); (3) Student Presentation: Organize “Course Ideology and Politics from Students' Perspectives” presentations, where students analyze societal issues using statistical methods; (4) Ethical Reflection: Discuss ethical considerations in data collection and analysis (e.g., data privacy, unbiased sampling in social surveys).

##### 3.3.2.4 Intuitive teaching for non-STEM majors

To reduce cognitive load, the course adopted simplified and visual approaches: (1) Pre-Theory Simulation: Use software (e.g., GeoGebra) for probability simulation before introducing formal theorems; (2) Tabular/Graphical Methods: Design standardized tables for conditional probability calculations and flowcharts for hypothesis testing; (3) Conceptual Prioritization: Focus on “why” (e.g., logic of probability) rather than “how” (e.g., complex computations); (4) Stepwise Scaffolding: Break complex problems into manageable steps with clear guidance (e.g., “Four-ball drawing” problem solved via tabular analysis).

##### 3.3.2.5 Detective Di Renjie problem-solving approach

Complex probability problems were framed as investigations with structured steps: (1) Problem Framing: Present scenarios as “cases” (e.g., “Determine the likelihood of suspect identification based on witness testimony”); (2) Evidence Collection: Guide students to extract key probability parameters and relationships from the scenario; (3) Logical Deduction: Apply probability rules using tabular/graphical methods to analyze the “case"; (4) Conclusion Verification: Validate results through peer discussion and instructor feedback. This approach was applied to 2-3 cases per chapter, enhancing cultural resonance and logical reasoning.

##### 3.3.2.6 Active learning strategies

Collaborative and interactive activities were systematically implemented: (1) Collaborative Problem-Solving: Small groups (3-4 students) tackle interdisciplinary statistical problems relevant to their majors; (2) Peer Teaching: Students take turns explaining key concepts (e.g., probability distributions) to peers, with instructor feedback; (3) Online Discussion Forums: Use Wenjuanxing platform for asynchronous discussions on case analyses and concept clarifications; (4) Reflective Journaling: Students document connections between course content and their major fields, with monthly instructor feedback.

#### 3.3.3 Assessment design

The assessment strategy was meticulously aligned with the defined learning outcomes, integrating both formative and summative components to support a developmental approach to learning.

Weekly quizzes served as low-stakes evaluations of foundational concepts, providing students with immediate feedback to reinforce understanding. Application projects required students to select datasets relevant to their academic disciplines, conduct rigorous analyses, and document their findings in structured written reports, thereby bridging theoretical knowledge with practical application. Oral presentations were incorporated to cultivate students' ability to articulate statistical reasoning clearly and persuasively to peer audiences, while reflective portfolios enabled them to document their learning trajectories and articulate interdisciplinary connections, fostering metacognitive awareness. The final examination, designed as a comprehensive evaluation, included both traditional problem-solving tasks and authentic application scenarios to assess mastery across the spectrum of learning outcomes.

Critically, formative assessments were characterized by detailed, constructive feedback and opportunities for revision and resubmission, reflecting OBE's core principle that learning is an iterative process requiring ongoing refinement and support.

### 3.4 Data collection

Data were collected through an online questionnaire administered during the final two weeks of the semester (weeks 15–16 of the 18-week semester). The recruitment period for this study spanned from 01/09/2024 to 31/01/2025. The timing allowed students to reflect on their full-semester experience while minimizing concerns about dropout. Participation was voluntary; students received no grade credit or penalty. The survey platform (Wenjuanxing) recorded responses anonymously. Completion time averaged 15–20 min.

Informed consent was obtained electronically from all participants prior to questionnaire completion. Participants were presented with a clear consent form outlining the study purpose, data use, guarantees of anonymity, and their right to withdraw without penalty. Consent was documented via the survey platform, which recorded a timestamp and digital confirmation of agreement. No minors were involved in this study, and all participants were adult undergraduate students (≥18 years old) capable of providing independent consent.

#### 3.4.1 Demographic characteristics

Table 3 presents the demographic characteristics of the sample by group. No significant differences were observed between the OBE and traditional groups in terms of gender distribution (χ^2^ = 0.32, *p* = 0.57) or major categories (χ^2^ = 2.15, *p* = 0.54), indicating baseline balance on key demographic variables.

### 3.5 Measurement instruments

The questionnaire comprised six constructs measured using multi-item scales adapted from validated instruments in educational and psychological research. All items used 5-point Likert scales (1=Strongly Disagree, 5=Strongly Agree) to ensure sufficient variance for SEM analysis.

**Course Mode (CM):** A single-item categorical variable indicating OBE-reformed (coded as 1) vs. traditional instruction (coded as 0).

**Knowledge Mastery (KM):** Six items adapted from Biggs (2003) measure students' self-reported perceived mastery of probability and statistics concepts. Sample item: “I can easily understand the basic concepts of probability and statistics taught in this course.”

**Statistical Application Ability (SAA):** Six items adapted from Heppner and Petersen (1982) measuring students' self-reported perceived ability to apply statistical knowledge to real-world problems. Sample item: “I can apply probability concepts to solve real-world problems such as simulating the spread of infectious diseases.”

**Cross-Disciplinary Thinking (CDT):** Six items adapted from Spelt et al. (2009) measuring students' self-reported perceived ability to integrate probability and statistics knowledge with other disciplinary domains. Sample item: “The course helped me see connections between probability and ideological education (e.g., national strategies).”

**Engagement (ENG):** Six items adapted from Fredricks et al. (2004) measuring students' behavioral and emotional engagement. Sample item: “I find the course activities engaging and enjoyable.”

**Self-efficacy (SE):** Six items adapted from Schwarzer and Jerusalem (1995) measuring students' confidence in their learning abilities. Sample item: “After this course, I feel confident in my ability to solve complex probability problems.”

#### 3.5.1 Adaptation and Validation of Measurement Instruments

Although all scales were adapted from well-validated instruments in the education and psychology literature, several contextual adaptations were necessary to align items with the specific probability and statistics course context and the Chinese educational setting. The adaptation process followed established guidelines for cross-cultural instrument adaptation (Beaton et al., 2000):

**Step 1: forward translation**. Original English items were translated into Chinese by two bilingual researchers independently.

**Step 2: expert review**. A panel of three subject-matter experts (two mathematics education researchers, one educational psychologist) reviewed the translated items for content validity, cultural appropriateness, and alignment with the constructs. Items were refined based on panel feedback.

**Step 3: cognitive pre-testing**. The adapted questionnaire was pilot-tested with 30 students (not included in the final sample) using think-aloud protocols to identify comprehension difficulties and ambiguous wording.

**Step 4: statistical validation**. The final questionnaire was evaluated for reliability (Cronbach's α, composite reliability) and validity (convergent and discriminant validity) in the full sample, as reported in Section 4.

We acknowledge that the adapted scales have not been subjected to independent psychometric validation (e.g., test-retest reliability, criterion validity against established instruments) beyond the procedures described above. This represents a limitation, and future research should conduct comprehensive validation studies of these context-specific measures (Willis, 2004).

#### 3.5.2 Behavioral indicators of engagement

In addition to self-reported engagement measures, objective behavioral indicators were collected to reduce common method bias:

1. Classroom Participation: Recorded as the number of voluntary contributions during in-class discussions and activities (range: 0-15 per student);

2. Online Interaction: Tracked through the learning management system, including forum posts, resource access frequency, and collaborative project contributions;

3. Assignment Completion Quality: Rated on a 5-point scale by two independent raters (κ = 0.87) based on adherence to requirements, depth of analysis, and application of course concepts.

These behavioral indicators were aggregated into a composite engagement index (α = 0.82) that showed significant correlations with self-reported engagement (*r* = 0.64, *p* < 0.001), providing convergent validity evidence. Importantly, this composite index served as an external validity check on the self-report ENG scale rather than as a separate construct in the PLS-SEM model; including it as a formative indicator would have required a distinct measurement specification beyond the scope of the present analysis. Future research is encouraged to formally integrate behavioral engagement indicators into structural models.

### 3.6 Objective academic performance measures

In addition to self-reported survey measures, we obtained two objective academic performance indicators from institutional records:

**Prior mathematics achievement**. Students' final examination scores in Calculus II (the prerequisite course), measured on a 100-point scale. This course was completed in the Fall 2023 semester, prior to enrollment in the probability and statistics course.

**Final examination performance**. Students' scores on the comprehensive final examination for the probability and statistics course, measured on a 100-point scale. Both groups took identical examinations administered under standardized conditions, ensuring measurement equivalence. The examination comprised computational problems (60%) and application scenarios (40%), assessing both procedural knowledge and conceptual understanding.

These objective measures complement the self-reported survey data by providing criterion validity evidence and addressing concerns about common method bias (Podsakoff et al., 2003).

### 3.7 Data analysis

Partial Least Squares Structural Equation Modeling (PLS-SEM) was conducted using SmartPLS (Hair et al., 2019). The choice of PLS-SEM over covariance-based SEM (CB-SEM) requires explicit justification, given that our model is primarily confirmatory in nature. We selected PLS-SEM for the following reasons:

First, PLS-SEM is recommended when the research objective includes both explanation and prediction of endogenous constructs (Hair et al., 2019; Sarstedt et al., 2022). Our study aims not only to test hypothesized mediating pathways but also to assess the predictive relevance (*Q*^2^) of the model for future observations, which PLS-SEM directly supports through blindfolding procedures.

Second, the inclusion of a single-item formative predictor (course modality, coded as a binary variable) is more naturally accommodated in PLS-SEM than in CB-SEM, which requires at least three indicators per latent construct for identification (Hair, 2014; Benitez et al., 2020).

Third, while CB-SEM relies on distributional assumptions (multivariate normality) and model-level fit indices (e.g., χ^2^, RMSEA), PLS-SEM uses bootstrapping for inference and evaluates predictive accuracy at the construct level (*R*^2^, *Q*^2^, *f*^2^). Given that our data showed some departure from multivariate normality (moderate kurtosis on several items), the distribution-free bootstrap inference in PLS-SEM is advantageous.

We acknowledge that PLS-SEM has been criticized for potentially overestimating path coefficients and providing less rigorous goodness-of-fit assessment than CB-SEM (Rönkkö and Evermann, 2013; Hair et al., 2019). To mitigate these concerns, we report multiple fit indicators (SRMR, NFI), effect sizes (*f*^2^), predictive relevance (*Q*^2^), and competing-model comparisons. We also emphasize that our PLS-SEM results are interpreted as evidence of associative patterns rather than confirmed causal mechanisms.

#### 3.7.1 Preliminary analysis

The preliminary analysis encompassed five methodological checks to ensure data integrity and comparability across groups. First, missing data patterns were examined to determine whether the missing-at-random (MAR) assumption held, with multiple imputation applied when conditions were satisfied. Second, potential outliers were identified using Mahalanobis distance calculations, followed by case-by-case examination of extreme values to assess their influence on statistical inferences. Third, distributional normality was evaluated through skewness and kurtosis statistics, supplemented by visual inspection of Q-Q plots. Fourth, common method bias was examined using Harman's single-factor test and variance inflation factor (VIF) diagnostics to assess collinearity among predictor variables. Finally, group equivalence on key demographic variables was verified through independent samples t-tests for continuous measures and chi-square tests for categorical variables, ensuring baseline comparability between the OBE reform and traditional teaching groups.

#### 3.7.2 Measurement model evaluation

Following Hair et al. (2019), the measurement model was evaluated using partial least squares structural equation modeling (PLS-SEM) criteria across three validity dimensions. For internal consistency reliability, both Cronbach's alpha and composite reliability (CR) coefficients were required to exceed 0.70, indicating acceptable levels of scale reliability. Convergent validity was established through two criteria: standardized factor loadings exceeding 0.70 on their respective constructs, and average variance extracted (AVE) values greater than 0.50, confirming that the constructs explained more variance in their indicators than measurement error. Discriminant validity was verified using both the Fornell-Larcker criterion, which requires the square root of AVE for each construct to exceed its correlations with all other constructs, and the heterotrait-monotrait ratio (HTMT), with values below 0.85 indicating adequate distinction between constructs.

##### 3.7.2.1 HTMT inference with bootstrap confidence intervals

In addition to reporting HTMT point estimates, we computed nonparametric bootstrap confidence intervals (2,000 resamples) for HTMT to support inference on discriminant validity. This procedure follows current recommendations that HTMT should be interpreted with confidence intervals rather than point estimates alone.

#### 3.7.3 Structural model testing

The hypothesized relationships were tested using partial least squares structural equation modeling (PLS-SEM) with bootstrapping procedures (5,000 resamples) to ensure robust statistical inference.

Model fit was evaluated through two criteria: the standardized root mean square residual (SRMR) required to be below 0.08, indicating acceptable approximation error, and the normed fit index (NFI) exceeding 0.90, suggesting good explanatory power relative to the null model. Path significance was assessed by examining standardized beta (β) coefficients alongside their 95% confidence intervals (CIs) to determine both direction and precision of effects. Effect sizes were quantified using Cohen's *f*^2^ statistics, with thresholds of 0.02, 0.15, and 0.35 representing small, medium, and large effects respectively.

Predictive relevance was verified through two metrics: *R*^2^ values greater than zero for endogenous constructs, indicating explained variance, and *Q*^2^ values exceeding zero in blindfolding procedures, confirming the model's out-of-sample predictive capacity.

#### 3.7.4 Multilevel modeling for clustered data (robustness check)

Since students were administratively assigned to intact class sections, observations were nested within classes (i.e., clustered data), which may violate the independence assumption of traditional statistical models. To account for potential non-independence in objective academic performance (final examination scores), hierarchical linear modeling (HLM; random-intercept models) was conducted as a robustness check, with students (Level 1) nested within class sections (Level 2).

First, an unconditional model was estimated to calculate the intraclass correlation coefficient (ICC), which quantifies the proportion of variance in final exam scores attributable to between-class differences. Second, a conditional model was fitted with grand-mean-centered Calculus II scores (to control for prior mathematical achievement) and instructional modality (OBE vs. traditional) as fixed effects:


$$\begin{array}{l} {\text{Final}}_{ij}=\beta_{0j}+\beta_{1}\left({\text{Calc2}}_{ij}-\bar{\text{Calc2}}\right)+\beta_{2}{\text{CM}}_{ij}+r_{ij}, \end{array}$$ (1)

$$\begin{array}{l} \beta_{0j}=\gamma_{00}+u_{0j}, \end{array}$$ (2)

where *r*_*ij*_ denotes Level-1 (student-level) residuals, *u*_0*j*_ represents random intercept deviations across class sections (Level 2), CM_*ij*_ is a dummy variable for instructional modality, and $\bar{\text{Calc2}}$ is the grand mean of Calculus II scores. To avoid unstable variance estimation, one class section with an extremely small cluster size (*n* = 1) was excluded from HLM analyses. This approach provides statistical inference robust to within-class dependence and complements the single-level ANCOVA results.

#### 3.7.5 Rationale for multiple analytical approaches

This study employs four analytical methods (ANCOVA, HLM, PSM, PLS-SEM), each addressing a distinct research question and a specific methodological concern. Rather than representing analytic redundancy, these methods serve complementary roles in a coherent triangulation strategy:

**ANCOVA** serves as the primary analysis for estimating the adjusted association between course modality and objective academic performance, controlling for the most salient covariate (prior mathematics achievement). **HLM** addresses the nested data structure (students within class sections), providing robustness to within-class dependence that ANCOVA cannot accommodate. **PSM** offers a complementary counterfactual framework that creates a balanced comparison group, reducing reliance on the linearity and homogeneity-of-slopes assumptions underlying ANCOVA (Austin, 2011). **PLS-SEM** examines a conceptually distinct question, which is to explore the psychological pathways linking OBE to perceived learning outcomes, using self-report data that cannot be analyzed by the objective-performance methods.

This multi-method design aligns with the principle of “methodological triangulation” (Denzin and N., 2012), whereby convergent findings across methods with different assumptions strengthen the overall evidence base. Each method compensates for the limitations of the others: ANCOVA assumes no unmeasured confounders; PSM relaxes linearity assumptions but requires overlap in propensity scores; HLM accounts for clustering but cannot address selection bias; PLS-SEM tests mediating mechanisms but relies on cross-sectional data. The convergence of positive OBE effects across all four approaches provides stronger evidence than any single analysis alone.

#### 3.7.6 Data anonymity and linkage constraint

The end-of-semester questionnaire was administered anonymously to comply with institutional ethical requirements and to encourage candid responses. This design decision, while enhancing data quality for self-reported measures, precludes linkage between individual survey responses and administrative academic records. Consequently, the study's two analytic strands operate on parallel but non-overlapping datasets: psychological mechanisms (PLS-SEM) are examined using survey data, while objective academic performance (ANCOVA, HLM, PSM) is examined using administrative records.

This separation constitutes a meaningful limitation. Specifically, we cannot empirically test whether individual students who reported higher engagement or self-efficacy also achieved higher examination scores. The two analytic strands therefore provide *convergent but independent* evidence: the objective performance analysis establishes that OBE is associated with superior examination outcomes at the group level, while the PLS-SEM analysis reveals the psychological pathways through which OBE relates to students' perceived learning processes. We interpret these parallel findings as mutually supportive but refrain from claiming that the psychological mechanisms identified in the SEM analysis causally explain the observed performance differences (Maxwell and Cole, 2007; Podsakoff et al., 2012).

Future research should employ designs that enable individual-level linkage between psychological measures and objective performance (e.g., coded but de-identified surveys) to directly test whether engagement and self-efficacy mediate the OBE–performance relationship at the individual level.

This critical constraint has important implications for causal inference. While we can demonstrate at the group level that (a) OBE is associated with better examination performance and (b) OBE is associated with higher reported engagement and self-efficacy, we cannot directly verify whether individual students with elevated engagement and self-efficacy scores were the same individuals who achieved higher academic performance. The inference that psychological mechanisms (engagement and self-efficacy) explain the observed performance differences therefore relies on a logic of parallel evidence rather than direct empirical linkage at the individual level. This limitation should temper interpretations of the dual-pathway model, as we cannot definitively establish that the psychological processes identified in the PLS-SEM analysis are the same processes that drive objective performance differences. Future studies employing coded (but de-identified) survey designs would enable individual-level mediation analysis, directly linking psychological constructs to objective performance outcomes and strengthening causal inference (Podsakoff et al., 2012).

#### 3.7.7 Dual-outcome analytic strategy

This study adopted a dual-outcome analytic approach to triangulate findings:

- Objective academic performance (final examination scores) was treated as an external criterion, analyzed via ANCOVA to estimate adjusted group differences associated with instructional modality.

- Latent constructs measured via self-report (e.g., engagement, self-efficacy, knowledge mastery, application ability, cross-disciplinary thinking) were analyzed using PLS-SEM to test hypothesized psychological mediating mechanisms.

This distinction aligns with methodological and theoretical considerations: objective exam scores capture summative learning outcomes under standardized assessment conditions, whereas the SEM framework focuses on students' perceived learning processes and outcomes that are critical for examining mediating mechanisms (Hair et al., 2019; Benitez et al., 2020). SEM results are thus interpreted as explanatory mechanisms that complement (rather than replace) the objective performance analysis.

### 3.8 Clarification of causal scope

Although the present study incorporates statistical adjustment techniques (ANCOVA and propensity score matching) to reduce bias arising from baseline differences, the design does not permit strong causal inference. All associations reported herein should therefore be interpreted as conditional associations rather than definitive causal effects. In particular, mediation analyses conducted using cross-sectional self-report data are intended to elucidate potential explanatory pathways rather than establish causal mechanisms.

It is important to emphasize that the observed associations between OBE instruction and learning outcomes may be influenced by unmeasured contextual factors (e.g., departmental teaching culture, peer learning dynamics) that cannot be fully controlled in quasi-experimental designs.

## 4 Results

This section presents the results of the study, beginning with a summary of the descriptive statistics for the key variables. Following this, the results are presented in two main analytic strands:

- Objective academic performance outcomes, analyzed through ANCOVA, HLM, and propensity score matching, to examine the impact of OBE on final exam scores.
- Psychological mechanisms (Engagement and Self-Efficacy) as mediators, analyzed using PLS-SEM to explore how these mechanisms influence learning outcomes.

First, we will review the descriptive statistics to provide an overview of the sample and key variables. Then, we will examine the impact of OBE on objective performance, followed by an analysis of the psychological pathways through which OBE influences student engagement, self-efficacy, and learning outcomes.

### 4.1 Descriptive statistics

Table 4 presents the descriptive statistics for all constructs by group. The mean values for the OBE group were consistently higher than those for the traditional group across all outcome variables, providing preliminary support for the effectiveness of OBE reform. Independent-samples *t*-tests confirmed significant differences between groups for all variables (all *p* < 0.001).

**Table 4:** Descriptive statistics by group.

Construct	OBE Group (*n* = 148)		Traditional Group (*n* = 173)		Total (*N* = 321)	
	Mean	SD	Mean	SD	Mean	SD
KM	3.616	0.682	3.115	0.738	3.344	0.740
ENG	3.627	0.639	3.152	0.691	3.370	0.694
SE	3.533	0.660	3.039	0.717	3.267	0.722
SAA	3.608	0.649	3.117	0.703	3.344	0.711
CDT	3.570	0.659	3.068	0.721	3.299	0.721

All items measured on 5-point Likert scales (1 = Strongly Disagree, 5 = Strongly Agree). Independent-samples t-tests indicate all group differences are statistically significant at *p* < 0.001, with the OBE group consistently scoring higher than the traditional group across all constructs.

### 4.2 Objective academic performance analysis

#### 4.2.1 Baseline comparison

Table 2 presents the comparison of prior mathematics achievement (Calculus II scores) between groups. The OBE group (*M* = 73.24, *SD* = 16.79) demonstrated significantly higher prior achievement than the traditional group (*M* = 63.22, *SD* = 18.76), *t*(319) = −5.02, *p* < 0.001, 95% CI [−13.95, −6.09], Cohen's *d* = −0.56. As detailed in the Methods section (Section 3.2), this baseline imbalance arose from administrative scheduling constraints prior to the availability of achievement data, rather than deliberate selection. The following analyses adjust for this difference using ANCOVA and propensity score matching to estimate the association between course modality and outcomes.

##### 4.2.1.1 Interpretation of baseline difference

This baseline difference warrants careful interpretation in light of the study's assignment procedure. As detailed in the Methods section, class sections were designated for OBE reform based on administrative scheduling constraints before prior achievement data became available. Therefore, the observed difference represents a chance imbalance arising from the natural academic heterogeneity across colleges and majors, rather than deliberate ability-based selection.

This interpretation is supported by several considerations:

1. **Temporal impossibility of selection bias:** The project team could not have selected higher-achieving students because their Calculus II grades were not yet available at the time of group assignment.
2. **Administrative basis of assignment:** Section assignment was driven by logistical factors (classroom scheduling, instructor availability) rather than student characteristics.
3. **Consistency with sampling variability:** Baseline imbalances of this magnitude can arise by chance when comparing non-equivalent groups drawn from different academic programs (Shadish, 2002).

Nonetheless, regardless of the source of baseline differences, statistical adjustment remains essential for valid comparisons between groups. ANCOVA controlling for prior achievement estimates the adjusted association between course modality and outcomes while accounting for pre-existing ability differences, under the assumption that no substantial unmeasured confounders are correlated with both assignment and outcomes (Murnane and Willett, 2010).

#### 4.2.2 Objective academic performance outcomes

Final examination scores were analyzed as an objective external performance criterion to validate whether the pedagogical effects observed in the SEM-based mechanism analysis were also reflected in standardized academic outcomes.

Table 5 presents the comparison of final examination scores. Students in the OBE group (*M* = 74.65, *SD* = 17.12) significantly outperformed those in the traditional group (*M* = 63.12, *SD* = 19.77) on the standardized final examination, *t*(319) = −5.52, *p* < 0.001, 95% CI [−15.64, −7.42]. The effect size (Cohen's *d* = −0.62) is considered medium to large according to conventional benchmarks (Cohen, 2013). However, given baseline differences in prior achievement, ANCOVA is essential to determine the extent to which this performance advantage is associated with the OBE instructional approach rather than pre-existing ability differences.

**Table 5:** Comparison of final examination scores by course modality.

Group	*n*	*M*	*SD*
Traditional	173	63.12	19.77
OBE Reform	148	74.65	17.12

Independent samples *t*-test: *t*(319) = −5.52, *p* < 0.001, 95% CI [−15.64, −7.42], Cohen's *d* = −0.62. Both groups completed identical examinations.

#### 4.2.3 Sensitivity analysis for unmeasured confounding

To quantify the robustness of the observed OBE effect to potential unmeasured confounding, we computed an E-value for the ANCOVA-adjusted effect (*B* = 6.89, *p* < 0.001) following VanderWeele and Ding (2017). The resulting E-value of 3.27 indicates that an unmeasured confounder would need to be associated with both course modality and final examination scores by a risk ratio of at least 3.27 simultaneously to fully explain the observed association. This magnitude is unlikely in educational settings (Phitayakorn et al., 2024).

Additionally, common method bias was assessed using the unmeasured latent method construct (ULMC) approach (Chen and Ding, 2025; Castillo et al., 2025). The method construct accounted for 12.3% of the total variance, well below the 25% threshold, suggesting that common method bias does not pose a severe threat to the validity of the structural model results.

#### 4.2.4 Comprehensive assessment of common method bias

Given the reliance on self-reported data for the PLS-SEM constructs, common method variance (CMV) represents a non-trivial concern. We employed three complementary approaches to assess and mitigate this risk:

**Procedural safeguards**. Following recommendations by Podsakoff et al. (2003, 2012), several procedural measures were implemented during survey design and administration: (a) the predictor variable (course modality) was determined by administrative records rather than self-report, eliminating same-source bias for the independent variable; (b) item ordering was varied across constructs to reduce consistency motifs; (c) response anchors were adapted across items where appropriate; and (d) anonymity was guaranteed to reduce social desirability bias.

**Harman's single-factor test**. An exploratory factor analysis of all 30 survey items yielded a first unrotated factor accounting for 38.7% of total variance, below the 50% threshold conventionally used to indicate severe CMV (Podsakoff et al., 2003). While this test has known limitations (low statistical power, insensitivity to moderate CMV), it provides preliminary evidence against a dominant method factor.

**Unmeasured latent method construct (ULMC) approach**. Following Liang et al. (2007); Chen and Ding (2025), we introduced an unmeasured common method factor into the PLS-SEM model. The average substantive variance explained by indicators was 0.687, while the average method variance was 0.123 (ratio ≈ 5.6: 1). The method factor accounted for 12.3% of total variance, well below the 25% threshold suggested by Williams et al. (2010). All substantive factor loadings remained significant after controlling for the method factor, and no indicator's method loading exceeded its substantive loading.

**Inclusion of objective performance data**. The study included objective final examination scores from institutional records as an external criterion. The significant OBE effect on objective performance (*B* = 6.89, *p* < .001), which is immune to self-report method bias, provides convergent evidence that complements the SEM-based findings.

Notwithstanding these assessments, we acknowledge that none of these approaches can entirely eliminate the risk of CMV in cross-sectional self-report data. The elevated inter-construct correlations (particularly ENG–SAA and SE–CDT) may be partially inflated by shared method variance, and structural path coefficients should be interpreted with this caveat in mind.

#### 4.2.5 ANCOVA: controlling for prior achievement

To isolate the effect of OBE reform from pre-existing differences in mathematical ability, we conducted ANCOVA with Calculus II scores as the covariate (Table 6). This analysis is particularly important given the significant baseline difference favoring the OBE group.

**Table 6:** ANCOVA results: effect of course modality on final examination controlling for prior achievement.

Predictor	B	SE	t	p	95% CI
Intercept	19.84	2.76	7.19	<0.001	[14.43, 25.25]
Prior achievement (calculus II)	0.70	0.04	17.50	<0.001	[0.63, 0.77]
Course modality (OBE vs. traditional)	6.89	1.35	5.12	<0.001	[4.24,9.54]

*R*^2^ = 0.573, *N* = 321, $\eta_{p}^{2}$ = 0.076

After controlling for prior mathematics achievement, course modality remained a significant predictor of final examination performance. The OBE group scored, on average, 6.89 points higher than the traditional group (*B* = 6.89, *SE* = 1.35, *t* = 5.12, *p* < 0.001, 95% CI [4.24, 9.54]). Prior achievement was a strong positive predictor (*B* = 0.70, *p* < 0.001), confirming both the relevance of this covariate and the importance of controlling for it. The full model explained 57.3% of variance in final examination scores ($\eta_{p}^{2}=0.076$ for the OBE effect, representing a medium effect).

Critically, the significant OBE effect after covariate adjustment demonstrates that the performance advantage of OBE students cannot be fully attributed to their higher baseline abilities. Even after statistically equating students on prior mathematical achievement, those who received OBE instruction scored nearly 7 points higher on the final examination. This finding strengthens the interpretation that the observed adjusted performance difference is not fully attributable to baseline ability differences, and is consistent with a robust association between OBE-oriented instruction and objective course performance under the study's adjustment strategy (Campbell and Stanley, 2015).

#### 4.2.6 HLM robustness check: accounting for class-level clustering

Because students were nested within class sections, we examined the extent of clustering in final examination scores. The unconditional (null) model estimated a between-class variance of τ_00_ = 40.229 and a within-class variance of σ^2^ = 341.079, yielding an ICC of 0.106. This indicates that approximately 10.6% of the total variance in academic performance was attributable to between-class differences, which justifies the use of multilevel modeling.

In the conditional random-intercept model (Table 7), the between-class variance decreased to τ_00_ = 1.987 and the within-class variance decreased to σ^2^ = 38.921. Importantly, course modality remained a significant predictor after accounting for class-level clustering (β = 2.324, SE=1.138, *p* = 0.041). These results indicate that the positive association between OBE instruction and objective academic performance is robust to within-class dependence. The reduction in variance components (from τ_00_ = 40.229 to 1.987 and from σ^2^ = 341.079 to 38.921) indicates that the fixed effects (prior achievement and course modality) explain substantial variance at both levels.

**Table 7:** Hierarchical linear modeling (random-intercept) results for final examination scores (*n* = 320, *J* = 8).

Fixed effects	Estimate	SE	95% CI	*p*
Intercept (at mean Calc2, CM=0)	67.244	0.805	[65.666, 68.821]	<0.001
Calc2 (grand-mean centered)	0.978	0.020	[0.939, 1.017]	<0.001
Course modality (CM; OBE=1)	2.324	1.138	[0.093, 4.554]	0.041
Random effects				
Between-class variance (τ_00_)	1.987			
Within-class variance (σ^2^)	38.921			

ICC = 0.106, computed from the unconditional (null) model as $\tau_{00}/\left(\tau_{00}+\sigma^{2}\right)=40.229/\left(40.229+341.079\right)$. Variance components shown above are from the conditional model.

#### 4.2.7 Propensity score matching: robustness check

As an additional robustness check, we implemented propensity score matching (PSM) using prior mathematics achievement as the matching variable. Given the significant baseline difference between groups (OBE students entered with higher Calculus II scores), PSM provides a complementary approach to ANCOVA by creating a matched sample with improved balance of covariates (Rosenbaum and Rubin, 1983; Austin, 2011).

One-to-one nearest-neighbor matching with a 5-point caliper yielded 72 matched pairs from the original sample of 321 students (Austin, 2011). The caliper restriction ensured that matched pairs had comparable prior achievement levels, though this conservative criterion excluded students at the extremes of the prior achievement distribution. After matching, the standardized mean difference in Calculus II scores between groups decreased to 0.08 (from 0.56), indicating successful balance on the matching variable (Austin and Stuart, 2015).

Analysis of the matched sample confirmed the main findings: OBE group students (*M* = 71.34, *SD* = 15.82) significantly outperformed their matched traditional group counterparts (*M* = 65.47, *SD* = 16.91) on final examinations, *t*(71) = 2.89, *p* = 0.005, Cohen's *d* = 0.36. Although the effect size in the matched sample (*d* = 0.36) was smaller than in the full sample (*d* = 0.62), this is expected given that PSM removes the portion of the effect attributable to baseline differences.

The consistency of significant OBE effects across analytical approaches, unadjusted comparison (*d* = 0.62), ANCOVA-adjusted (*B* = 6.89, $\eta_{p}^{2}=0.076$), and propensity-matched (*d* = 0.36), strengthens confidence in the robustness of the finding that OBE instruction enhances objective academic performance beyond what would be predicted from students' prior mathematical abilities alone.

Having established that OBE instruction is associated with improved objective academic performance after multiple robustness checks, we next examine the psychological mechanisms underlying these differences using structural equation modeling.

### 4.3 Psychological mechanism analysis (PLS-SEM)

#### 4.3.1 Measurement model assessment

The preceding subsections (Sections 4.1–4.2.7) established the objective performance basis of the OBE effect using administrative exam records. The following sections turn to the PLS-SEM analysis of self-reported constructs, which aims to elucidate the psychological mechanisms (engagement and self-efficacy) through which OBE influences perceived learning outcomes. This two-stranded analytic approach is explained in the Dual-Outcome Analytic Strategy subsection of the Methodology (Section 3).

##### 4.3.1.1 Internal consistency reliability

Table 8 presents the reliability indices for all constructs. Cronbach's alpha values ranged from 0.882 to 0.906, exceeding the recommended threshold of 0.70 (Tavakol and Dennick, 2011). Composite reliability (CR) values, measured by Dillon-Goldstein's rho, ranged from 0.883 to 0.907, also exceeding the 0.70 threshold. These results indicate excellent internal consistency reliability.

**Table 8:** Construct reliability and validity.

Construct	Items	Cronbach's α	CR	AVE	$\sqrt{\text{AVE}}$
ENG	6	0.887	0.888	0.640	0.800
KM	6	0.903	0.905	0.673	0.821
SE	6	0.906	0.907	0.680	0.825
SAA	6	0.882	0.883	0.629	0.793
CDT	6	0.894	0.895	0.654	0.808

CR, Composite Reliability (Dillon-Goldstein's rho); AVE, Average Variance Extracted.

##### 4.3.1.2 Convergent validity

Convergent validity was assessed through factor loadings and Average Variance Extracted (AVE). As shown in Table 9, all factor loadings exceeded the recommended threshold of 0.70 (Hair et al., 2019), ranging from 0.773 to 0.851. AVE values for all constructs exceeded 0.50 (Table 8), indicating that the constructs explain more than half of the variance in their indicators. These results support adequate convergent validity.

**Table 9:** Factor loadings.

Item	Loading	Item	Loading	Item	Loading	Item	Loading	Item	Loading
ENG1	0.800	SE1	0.841	SAA1	0.795	KM1	0.836	CDT1	0.822
ENG2	0.837	SE2	0.806	SAA2	0.816	KM2	0.803	CDT2	0.823
ENG3	0.790	SE3	0.801	SAA3	0.780	KM3	0.823	CDT3	0.813
ENG4	0.785	SE4	0.819	SAA4	0.773	KM4	0.842	CDT4	0.788
ENG5	0.778	SE5	0.829	SAA5	0.788	KM5	0.827	CDT5	0.798
ENG6	0.808	SE6	0.851	SAA6	0.808	KM6	0.791	CDT6	0.816

All loadings significant at *p* < 0.001.

##### 4.3.1.3 Discriminant validity

Discriminant validity was assessed using multiple complementary criteria in line with contemporary best practices (Henseler et al., 2015; Voorhees et al., 2016): the Fornell-Larcker criterion and the heterotrait-monotrait (HTMT) ratio with bootstrap confidence intervals.

1.Fornell-Larcker Criterion As shown in Table 10, the square root of average variance extracted (AVE) for each construct (bold diagonal values) exceeded its bivariate correlations with all other constructs (below-diagonal values), satisfying the Fornell-Larcker criterion for discriminant validity.

**Table 10:** Discriminant validity: Fornell-Larcker criterion and HTMT ratios.

Construct	ENG	KM	SAA	SE	CDT
Engagement (ENG)	**0.800**	0.651	0.698	0.633	0.551
Knowledge mastery (KM)	0.585	**0.821**	0.679	0.558	0.423
Statistical application (SAA)	0.619	0.607	**0.793**	0.554	0.472
Self-efficacy (SE)	0.568	0.505	0.494	**0.825**	0.808
Cross-disciplinary (CDT)	0.492	0.380	0.419	0.728	**0.808**

Diagonal values (bold) = square roots of AVE; below-diagonal values = inter-construct Pearson correlations; above-diagonal values = heterotrait-monotrait (HTMT) ratios.

2.HTMT ratio with bootstrap confidence intervals

To provide a more stringent test of discriminant validity (Henseler et al., 2015), we computed the HTMT ratio and nonparametric bootstrap 95% confidence intervals (5,000 resamples). Table 11 presents the HTMT values and their confidence intervals for all construct pairs.

**Table 11:** HTMT ratios with 5,000-bootstrap 95% confidence intervals.

Construct pair	HTMT	95% CI (Lower)	95% CI (Upper)
CDT–ENG	0.551	0.464	0.632
CDT–SE	0.808	0.754	0.854
ENG–SE	0.633	0.548	0.710
KM–CDT	0.423	0.323	0.519
KM–ENG	0.651	0.566	0.728
KM–SAA	0.679	0.600	0.750
KM–SE	0.558	0.471	0.639
SAA–CDT	0.472	0.365	0.569
SAA–ENG	0.698	0.625	0.765
SAA–SE	0.554	0.457	0.638

All HTMT point estimates fell below the conservative threshold of 0.85, with the highest value observed for CDT–SE (HTMT = 0.808, 95% CI [0.754, 0.854]). Several other construct pairs showed moderate HTMT values, including SAA–ENG (HTMT = 0.698, 95% CI [0.625, 0.765]) and KM–SAA (HTMT = 0.679, 95% CI [0.600, 0.750]). Although all point estimates satisfied the 0.85 criterion, the upper bound of the bootstrap confidence interval for CDT–SE (0.854) approached the threshold, suggesting potential construct proximity between self-efficacy and cross-disciplinary thinking when measured via self-report.

3. Interpretation of discriminant validity results While the Fornell-Larcker criterion indicated acceptable discriminant validity, the HTMT results suggest partial conceptual overlap among the self-reported constructs. This pattern is not unexpected given: (1) the theoretical proximity of engagement, self-efficacy, and higher-order learning outcomes (SAA, CDT); and (2) the potential for a global positivity bias in self-report measures (i.e., students with positive course perceptions tend to rate multiple domains highly).

Despite the elevated HTMT values, the constructs were retained as distinct for three reasons: (1) strong theoretical justification for their conceptual separability; (2) acceptable composite reliability and AVE values (reported elsewhere); and (3) consistent with prior research on self-reported motivational and learning outcome constructs. To address this limitation, subsequent structural model coefficients are interpreted as strong latent associations among closely related perceptions (rather than sharply separable psychological constructs). We further mitigated this issue by: (a) testing competing structural specifications to evaluate whether large coefficients were driven by parsimony constraints; and (b) recommending future measurement refinement, such as incorporating behaviorally anchored indicators for application and interdisciplinary transfer (e.g., learning analytics, observed participation) or modeling a higher-order perceived learning outcome factor to account for shared variance among related constructs. These approaches would provide a more stringent test of discriminant validity and help disentangle the unique contributions of each construct.

##### 4.3.1.4 Addressing construct proximity concerns

The elevated correlations and HTMT values between certain construct pairs warrant discussion. We offer three interpretations:

First, **theoretical proximity with conceptual distinctiveness**: Engagement and self-efficacy are theoretically related constructs within social cognitive theory, as engagement experiences contribute to self-efficacy development, and self-efficacy influences willingness to engage (Bandura, 2023; Skinner et al., 2009). Similarly, perceived application ability and cross-disciplinary thinking both reflect higher-order learning outcomes that naturally co-develop. High correlations may reflect genuine psychological covariation rather than measurement redundancy.

Second, **common learning context effects**: All constructs were measured within the same course context, and students who experience positive learning outcomes in one dimension likely experience positive outcomes in others. This “rising tide” effect is substantively meaningful rather than artifactual (Marsh and Craven, 2006).

Third, **self-report method effects**: As acknowledged in the Limitations, self-reported perceptions may share method variance that inflates inter-construct correlations. Including objective academic performance measures (final examination scores) provides an external criterion that partially addresses this concern.

Although the Fornell–Larcker criterion was satisfied, the HTMT results (with the CDT–SE upper bound of 0.854 approaching the 0.85 threshold) indicate **non-trivial construct proximity** for certain pairs. Therefore, the latent variables should be interpreted as closely related perceived dimensions rather than sharply separable psychological constructs, and structural coefficients are discussed with this measurement limitation in mind.

##### 4.3.1.5 Theoretical basis for treating constructs as distinct

The high correlations between certain construct pairs (particularly SE–CDT: *r* = 0.728; ENG–SAA: *r* = 0.619) raise the question of whether these represent genuinely distinct constructs or overlapping facets of a broader latent factor. We provide three lines of justification for retaining the five-construct structure:

**Nomological distinctiveness**. Each construct occupies a distinct position in the theoretical model and is expected to relate differently to other variables. Self-efficacy (a motivational belief about capability) and cross-disciplinary thinking (a cognitive outcome reflecting knowledge integration) are conceptually distinct even when empirically correlated. The competing-model analysis (Table 1) demonstrates that when both ENG and SE are allowed to predict all three outcomes, their differential predictive patterns are preserved (ENG is a stronger predictor of KM and SAA; SE contributes uniquely to CDT alongside ENG), supporting their conceptual separability.

**Supplementary HTMT**_**2**_ **analysis**. Following (Roemer et al., 2021), we computed the geometric-mean-based HTMT_2_ statistic, which is more robust to violations of the tau-equivalence assumption. All HTMT_2_ values remained below 0.85, with the highest value of 0.796 for CDT–SE, providing additional support for discriminant validity.

**Higher-order factor alternative**. To directly test whether the three outcome constructs (KM, SAA, CDT) are empirically distinguishable from a general “perceived learning outcomes” factor, we estimated a competing model with a second-order factor (Competing Model B, Table 1). The results show that this model fits well (*R*^2^ = 0.865) but obscures meaningful differential relationships between mediators and specific outcomes. This suggests that while the outcomes share variance, a differentiated model provides greater theoretical insight.

Despite these justifications, we emphasize that the elevated construct proximity, particularly for SE CDT, represents a genuine measurement limitation. Our interpretations of structural paths involving these constructs are accordingly conservative, and we recommend that future research develop more differentiated measures incorporating behaviorally anchored and performance-based indicators (Henrie et al., 2015; Willis, 2004).

#### 4.3.2 Structural model assessment

##### 4.3.2.1 Collinearity assessment

Prior to hypothesis testing, collinearity among predictor constructs was assessed. All Variance Inflation Factor (VIF) values were below 3.0 (ranging from 1.234 to 2.156), indicating that collinearity was not a concern (Sarstedt et al., 2022).

##### 4.3.2.2 Path coefficients and hypothesis testing

Table 12 presents the results of hypothesis testing using bootstrapping with 5,000 resamples. All five direct effect hypotheses (H1, H2, H3, H4, and H7) were supported.

**Table 12:** Structural model results: direct effects.

Hypothesis	Path	β	SE	t-value	95% CI	*p*-value	Decision
H1	CM → ENG	0.263	0.051	5.181	[0.180, 0.347]	<0.001	Supported
H2	CM → SE	0.276	0.052	5.316	[0.190, 0.360]	<0.001	Supported
H3	ENG → KM	0.587	0.039	15.016	[0.518, 0.647]	<0.001	Supported
H4	ENG → SAA	0.621	0.033	18.582	[0.562, 0.671]	<0.001	Supported
H7	SE → CDT	0.731	0.025	29.367	[0.686, 0.767]	<0.001	Supported

β, standardized path coefficient; SE, standard error; CI, confidence interval.

Bootstrap samples = 5,000.

##### 4.3.2.3 Interpreting unusually large standardized path coefficients and model robustness

Two standardized paths in the focal model were unusually large (ENG → SAA; SE → CDT). Such magnitudes are uncommon in educational psychology studies and may arise when (a) endogenous outcomes are specified with a single dominant antecedent, (b) constructs are measured concurrently via self-report, and (c) discriminant validity is strained for conceptually adjacent constructs.

Consistent with this concern, the HTMT analysis with bootstrap confidence intervals indicated substantial construct overlap for ENG–SAA, ENG–CDT, and SE–CDT (Table 11). Under this measurement context, large standardized coefficients should be interpreted primarily as strong latent associations among students' concurrent perceptions rather than isolated causal effects.

To evaluate whether the large coefficients are driven by model parsimony rather than theoretically unique pathways, we conducted robustness checks using theoretically plausible competing specifications: (1) allowing both ENG and SE to predict all three outcomes (KM, SAA, CDT) and comparing changes in path magnitudes; and (2) testing an alternative higher-order factor model in which KM, SAA, and CDT load onto a second-order “perceived learning outcomes” factor. The substantive conclusion regarding the positive association between OBE modality and students' reported learning processes remained stable, while the extreme magnitudes of single-antecedent paths were attenuated in the less restrictive models. Detailed results are provided in the Supplementary Material.

##### 4.3.2.4 Coefficient of determination (*R*^2^) and effect size (*f*^2^)

The model explained substantial variance in all endogenous constructs. As shown in Table 13, *R*^2^ values ranged from 0.203 to 0.804, indicating moderate to substantial explanatory power according to the criteria suggested by Cohen (2013) statistical. Effect sizes were calculated to assess the practical significance of the relationships. According to Lakens and Caldwell (2021), *f*^2^ values of 0.02, 0.15, and 0.35 represent small, medium, and large effects, respectively. As shown in Table 13, most relationships exhibited medium to large effect sizes.

**Table 13:** Coefficient of determination (*R*^2^) and effect sizes (*f*^2^).

Construct	*R* ^2^	$R_{adj}^{2}$	Interpretation	Path	*f* ^2^	Interpretation
ENG	0.203	0.202	Moderate	MODE → ENG	0.255	Medium
SE	0.211	0.210	Moderate	MODE → SE	0.267	Medium
KM	0.740	0.739	Substantial	ENG → KM	2.846	Large
SAA	0.804	0.804	Substantial	ENG → SAA	4.101	Large
CDT	0.759	0.759	Substantial	SE → CDT	3.149	Large

*R*^2^ interpretation follows Cohen (2013): 0.02 = weak, 0.13 = moderate, 0.26 = substantial; *f*^2^ interpretation: 0.02 = small, 0.15 = medium, 0.35 = large.

Beyond statistical significance, the practical significance of the observed effects warrants discussion. The CM → ENG and CM → SE path coefficients (β = 0.265 and 0.276, respectively) represent small-to-medium effects according to Cohen (2013), indicating that while OBE instruction is associated with meaningful improvements in psychological processes, substantial individual variability remains unexplained by course modality alone. The corresponding *f*^2^ values (0.255 and 0.267; Table 13) fall in the medium range, confirming that these effects, while not trivially small, are also not overwhelmingly large. This is consistent with the general finding in educational intervention research that pedagogical innovations typically produce small-to-medium effects (Hattie, 2008; Kraft, 2020).

For the objective performance analysis, the PSM-adjusted effect size (*d* = 0.36) is particularly informative, as it removes the influence of baseline ability differences. According to Kraft (2020), effect sizes of *d* ≥ 0.20 in educational settings represent practically meaningful improvements, placing our finding in the range of consequential educational effects.

##### 4.3.2.5 Predictive relevance (*Q*^2^)

Stone-Geisser's *Q*^2^ values were computed using the blindfolding procedure to assess the model's predictive relevance. All *Q*^2^ values exceeded zero (KM: 0.544, SAA: 0.621, CDT: 0.566, ENG: 0.158, SE: 0.153), indicating satisfactory predictive relevance.

##### 4.3.2.6 Robustness checks: competing structural specifications (composite-score path models)

Table 1 summarizes competing specifications estimated using standardized composite scores. In the focal (single-antecedent) specification, ENG strongly predicted KM (β = 0.860) and SAA (β = 0.897), and SE strongly predicted CDT (β = 0.871). In Competing Model A (cross-links), coefficients attenuated: ENG → SAA decreased to β = 0.710, and SE → CDT decreased to β = 0.192, while ENG emerged as a strong predictor of CDT (β = 0.754). In Competing Model B, both ENG (β = 0.691) and SE (β = 0.214) significantly predicted the higher-order perceived learning outcomes factor (PLO). These results indicate that unusually large coefficients in the focal model are partly attributable to parsimony constraints under concurrent self-report measurement.

#### 4.3.3 Mediation analysis

Table 14 presents the results of mediation analysis for H5, H6, and H8. All three mediation hypotheses were supported, with significant indirect effects and confidence intervals that did not include zero.

**Table 14:** Mediation analysis results.

Hyp.	Mediation path	a	b	Indirect	Direct	Total	95% CI	VAF
H5	CM → ENG → KM	0.263^**^	0.538^**^	0.142^**^	0.170^**^	0.311^**^	[0.080, 0.203]	45.7%
H6	CM → ENG → SAA	0.263^**^	0.577^**^	0.152^**^	0.155^**^	0.307^**^	[0.087, 0.217]	49.5%
H8	CM → SE → CDT	0.274^**^	0.708^**^	0.194^**^	0.068^**^	0.262^**^	[0.116, 0.272]	74.0%

^*^ *p* < 0.01,

** *p* < 0.001

VAF, Variance Accounted For (indirect effect / total effect), reported as the proportion of the total association accounted for by the indirect path.

The Variance Accounted For (VAF) values indicate that a substantial proportion of the total association between course modality and students' perceived learning outcomes is explained by indirect paths through engagement and self-efficacy. These results suggest that engagement and self-efficacy represent statistically meaningful explanatory pathways linking OBE-oriented instruction with self-reported learning outcomes. However, given the cross-sectional nature of the data, where mediators and outcomes were measured simultaneously at the end of the semester, these indirect effects should be interpreted as statistical indirect associations consistent with the hypothesized mediating pathways, rather than evidence of causal mediation in the strict temporal sense (Maxwell and Cole, 2007). The term “mediation” is used throughout this paper in its statistical sense (i.e., significant indirect effect via bootstrapping) and should not be interpreted as establishing that engagement or self-efficacy causally transmit the effect of OBE to learning outcomes. Establishing causal mediation would require, at minimum, temporal separation between the predictor, mediators, and outcomes, and ideally experimental manipulation of the mediators themselves (Bullock et al., 2010).

It is worth noting that the *b*-path coefficients in the mediation model (ENG → KM = 0.538; ENG → SAA = 0.577; SE → CDT = 0.708) are slightly lower than the corresponding estimates in the direct-effects model (Table 12: ENG → KM = 0.587; ENG → SAA = 0.621; SE → CDT = 0.731). This attenuation is methodologically expected because the mediation model includes a direct path from course modality (CM) to each outcome, which absorbs part of the shared variance. Both sets of estimates are internally consistent within their respective model specifications, and the pattern of attenuation aligns with partial mediation.

## 5 Discussion

The findings are consistent with a positive association between OBE and both objective performance and psychological outcomes. Engagement and self-efficacy were identified as key mediators in this relationship. These results contribute to the theoretical understanding of how OBE fosters student learning and provide practical implications for curriculum design.

### 5.1 Summary of findings

This study investigated the effectiveness of OBE reform in probability and statistics courses within the New Liberal Arts context. All eight hypotheses received empirical support, providing evidence consistent with the proposed dual-pathway model linking OBE to perceived learning outcomes through engagement and self-efficacy. However, given the cross-sectional measurement of mediators and outcomes, these findings should be understood as identifying statistically significant indirect associations rather than confirming causal mediation processes.

The OBE course modality demonstrated significant positive effects on both student engagement (β = 0.265, *p* < 0.001) and self-efficacy (β = 0.276, *p* < 0.001), confirming H1 and H2. These medium effect sizes indicate that the pedagogical innovations implemented in the OBE course, including cultural integration, contemporary cases, and interactive activities, meaningfully enhanced students' psychological investment and confidence.

The engagement pathway showed strong associations with both knowledge mastery (H3: β = 0.587, *p* < 0.001) and statistical application ability (H4: β = 0.621, *p* < 0.001), and the self-efficacy pathway showed a strong association with cross-disciplinary thinking (H7: β = 0.731, *p* < 0.001).

Given that SAA and CDT are each modeled with a single antecedent and all three outcomes are measured via self-reports, these large coefficients should be interpreted as strong latent associations rather than definitive causal effects. The relatively high correlations between ENG and SAA, and between SE and CDT (Table 10), suggest that these construct pairs may be conceptually close in students' perceptions, which we address further in Section 5.3 and the Remaining Limitations.

### 5.2 Theoretical implications

First, this study shifts the focus of OBE research to mathematics education within the liberal arts context. Previous studies have primarily focused on engineering and professional education (Sobri et al., 2025; Mahrishi et al., 2025), whereas our results indicate that OBE principles are equally applicable to foundational mathematics courses serving diverse student populations. This extends the generalizability of OBE theory beyond professional disciplines, particularly relevant given the New Liberal Arts' focus on interdisciplinary competencies and data literacy.

Second, our results shed light on the psychological factors associated with the relationship between OBE and learning outcomes. The notable mediating associations of engagement (H5, H6) and self-efficacy (H8) align with Bandura (2023)'s social cognitive theory, which emphasizes the interplay between environmental structures and psychological beliefs in shaping behavior. This complements Fredricks et al. (2004)'s engagement framework by demonstrating how outcome-oriented curriculum design can foster multidimensional engagement in mathematics education.

Third, the distinct mediating patterns observed align with Bloom's taxonomy of learning objectives (Carter, 1985; Lee-Robbins and Adar, 2022). Engagement is primarily associated with foundational (KM) and application (SAA) outcomes (*VAF* = 45.7% and 49.5%, respectively), whereas self-efficacy is more strongly associated with higher-order cross-disciplinary thinking (*VAF* = 74.0%). This pattern supports the notion that higher-order learning requires both behavioral engagement and psychological empowerment (Zimmerman, 1995).

Fourth, the substantial *R*^2^ values for outcome variables (KM: 74.0%, SAA: 80.4%, CDT: 75.9%) exceed typical explanatory power in educational intervention studies (Hattie, 2008), indicating that the combination of OBE pedagogy with strategies to enhance engagement and self-efficacy represents a particularly effective approach to mathematics education reform.

Fifth, a distinctive theoretical contribution of this study lies in the differentiation of outcome dimensions and their corresponding mediating pathways. Prior OBE research has typically used undifferentiated achievement measures (Pinilla et al., 2021; Sobri et al., 2025). By distinguishing among knowledge mastery, statistical application ability, and cross-disciplinary thinking, we demonstrate that different learning outcomes may be associated with different psychological pathways. This finding has important implications for intervention design: if foundational outcomes are primarily linked to engagement, while higher-order integrative outcomes are primarily linked to self-efficacy, then pedagogical strategies should be tailored to target the specific mechanism most relevant to the intended outcome.

Rather than establishing causal mechanisms, the present findings suggest that student engagement and self-efficacy constitute theoretically grounded, statistically supported explanatory pathways associated with students' perceived learning outcomes in outcome-oriented probability and statistics instruction. These pathways clarify how OBE-aligned instructional practices relate to students' learning experiences within the New Liberal Arts context.

### 5.3 Practical implications for curriculum design and instructional practice

Based on the dual-pathway model and empirical findings, we propose actionable recommendations for educators, curriculum designers, and higher education administrators implementing OBE reforms in mathematics and cross-disciplinary courses:

#### 5.3.1 Design student-centered learning activities to enhance engagement

The significant mediating effect of engagement, with variance accounted for (VAF) of 45.7% for Knowledge Mastery (KM) and 49.5% for Statistical Application Ability (SAA), underscores the imperative to integrate culturally pertinent and contextually authentic activities into probability and statistics courses. For instance, cultural elements can be incorporated, such as Chinese proverbs like “Slander repeated three times can make even a loving mother doubt her child” to elucidate conditional probability, and detective stories to enhance logical reasoning, thereby bridging abstract mathematical concepts with students' cultural backgrounds (Li and Li, 2024). Contemporary real-world cases, such as COVID-19 epidemic modeling, social media data analysis, and legal probability applications, should be embedded to align with students' disciplinary majors and career aspirations (Clark, 2025; Dinov et al., 2008). Additionally, active learning strategies, including collaborative problem-solving, peer teaching, and online discussion forums, should be adopted to foster behavioral, emotional, and cognitive engagement (Mengesha et al., 2024; Afroze and Shafi, 2024).

#### 5.3.2 Implement scaffolded instruction to build self-efficacy

Given that self-efficacy accounts for 74.0% of the mediating effect of Outcome-Based Education (OBE) on cross-disciplinary thinking, instructors should prioritize strategies to boost students' confidence in applying mathematical knowledge. Complex learning outcomes should be decomposed into manageable milestones, each accompanied by explicit assessment criteria. Additionally, formative feedback and opportunities for revision should be provided to facilitate the creation of mastery experiences (Bandura, 2023; Zahidi and Ong, 2023). Scaffolding techniques, such as using tabular and graphical methods for probability calculations and adopting simulation-based learning before formal theory is introduced, can help reduce cognitive load, particularly for students from non-STEM majors (Almulla, 2023; Xu et al., 2024). Moreover, the integration of success stories involving interdisciplinary applications, for example, statistical analysis in social policy and probability models in ethical decision-making, can offer students vicarious learning experiences (Zimmerman, 2000; Qiu et al., 2025).

#### 5.3.3 Foster cross-disciplinary thinking through self-efficacy enhancement

To attain the objectives of the New Liberal Arts in dismantling disciplinary barriers, several strategies are worth implementing. Firstly, transferable skills should be taught explicitly, guiding students in identifying the links between probability and statistics and their respective major fields. For instance, social science students could be guided to analyze survey data, while humanities students could be encouraged to evaluate statistical claims presented in the media (Spelt et al., 2009; Kim et al., 2024). Secondly, interdisciplinary projects should be carefully crafted to merge mathematical reasoning with domain-specific knowledge. Examples include analyzing national statistical achievements for ideological education or assessing social equity using statistical data (Gao, 2021; Jing and Tan, 2024). Thirdly, targeted assistance, including peer mentoring and personalized learning plans, should be provided to students with low initial self-efficacy to foster boundary-crossing thinking (Kaufmann et al., 2022; Azzarello et al., 2025).

#### 5.3.4 Align assessment with learning outcomes to reinforce the OBE cycle

Assessment design should be closely aligned with intended learning outcomes to support the iterative OBE process. Low-stakes weekly quizzes can be used to reinforce foundational knowledge and provide timely feedback (Biggs, 2003; Hair et al., 2019). Application projects should require students to select discipline-specific datasets, conduct analyses, and present findings in both written and oral formats (Heppner and Petersen, 1982; Seifert et al., 2008). Reflective portfolios help students document their learning trajectories and articulate interdisciplinary connections, thereby fostering metacognitive awareness (Fredricks et al., 2004; Henrie et al., 2015). Comprehensive final examinations should balance procedural knowledge (60%) and authentic application scenarios (40%) to assess multiple learning outcomes (Batanero and Álvarez-Arroyo, 2024; Jiang et al., 2019).

#### 5.3.5 Adapt to diverse student populations and institutional contexts

For liberal arts students, the pedagogical focus should be shifted toward conceptual understanding rather than computational mechanics, employing intuitive methods to elucidate complex theoretical concepts (Li and Li, 2024; Cheng, 2022). For STEM students, OBE reforms can be extended by incorporating advanced interdisciplinary applications, such as integrating machine learning techniques or quantitative modeling into natural science contexts (Mahrishi et al., 2025; Sobri et al., 2025). For public universities and other diverse institutional settings, the cultural and contextual components of the curriculum should be adapted to align with students' backgrounds, while maintaining the fundamental objective of linking abstract knowledge with meaningful real-world applications (Hofstede, 2011; Franco et al., 2024).

#### 5.3.6 Address OBE implementation challenges with targeted mitigation strategies

While the aforementioned strategies highlight the potential of OBE, successful implementation requires proactive management of practical challenges. Institutions and instructors should anticipate and address key barriers through tailored solutions:

##### 5.3.6.1 Instructor capacity building

OBE requires a paradigm shift from content delivery to learning facilitation. Institutions should provide targeted professional development, including workshops on constructive alignment, formative assessment design, and student-centered pedagogical techniques (Jaya et al., 2025; Shaheen, 2019). Peer mentoring programs (e.g., pairing OBE-experienced instructors with novices) can also accelerate capacity building.

##### 5.3.6.2 Resource and time optimization

Developing authentic cases, designing scaffolded activities, and providing individualized feedback demand additional time and resources. Collaborative curriculum development (e.g., interdisciplinary instructor teams co-designing cases) and integration of digital tools (e.g., simulation software for probability modeling, online feedback platforms) can alleviate workload (Xu et al., 2024; Syeed et al., 2022).

##### 5.3.6.3 Student adaptation support

Students accustomed to traditional lecture-based instruction may initially struggle with OBE's emphasis on autonomy and active participation. Scaffolded transition strategies include clear rubrics for expected outcomes, guided practice in collaborative problem-solving, and peer learning groups to build confidence (Almulla, 2023; Kaufmann et al., 2022).

##### 5.3.6.4 Institutional policy alignment

Sustainable OBE reform requires institutional support beyond individual courses. Policies such as flexible class scheduling, recognition of OBE-related workload in promotion criteria, and dedicated funding for curriculum development can create an enabling environment (Gao, 2021; Jing and Tan, 2024).

These mitigation strategies address the practical realities of OBE implementation, ensuring that the theoretical benefits of the dual-pathway model translate into sustained improvements in teaching and learning outcomes.

### 5.4 Robustness, causal inference, and methodological considerations

#### 5.4.1 Design-level safeguards

A central concern in quasi-experimental research is whether observed effects reflect genuine instructional impacts rather than pre-existing group differences. Three design features of the present study mitigate this concern.

##### 5.4.1.1 Administrative random assignment

Class sections were designated for OBE reform based on administrative scheduling constraints *before* students' prior achievement data (Calculus II scores) became available. This temporal separation establishes that assignment was **independent of the primary confounding variable**, thereby satisfying a key assumption for unbiased causal inference (Shadish, 2002; Steiner et al., 2010). While the observed baseline difference (Cohen's *d* = −0.56) might initially suggest selection bias, the assignment procedure rules out deliberate ability-based sorting. The difference instead reflects natural variation in academic composition across colleges and majors, which is sampling variability rather than systematic selection (Rubin, 2008).

##### 5.4.1.2 Same-instructor, same-semester design

Both groups were taught by the same instructor during the same semester, eliminating confounding due to instructor effects, temporal factors, and examination difficulty. This design feature is particularly valuable because instructor quality is often the largest source of variation in educational outcomes (Hanushek, 2011; Chetty et al., 2014).

##### 5.4.1.3 Ruling out alternative explanations

Ability-based selection is ruled out by the temporal assignment sequence; instructor effects by the same-instructor design; temporal/cohort effects by the same-semester structure; and examination difficulty by using identical assessments. Hawthorne effects are unlikely, as both groups were enrolled in the same course and received no differential experimental attention.

#### 5.4.2 Statistical adjustment strategies

##### 5.4.2.1 ANCOVA

Controlling for prior achievement yielded a significant OBE effect (*B* = 6.89, *p* < 0.001, $\eta_{p}^{2}=0.076$)(see Table 6), demonstrating that the intervention effect persists after adjustment for the most plausible confound. Under the assumption that assignment was independent of prior achievement (established by the temporal sequence) and no unmeasured confounders exist, ANCOVA provides an approximately unbiased estimate of the average treatment effect (Murnane and Willett, 2010).

##### 5.4.2.2 Propensity score matching (PSM)

As a complementary robustness check, one-to-one nearest-neighbor PSM (5-point caliper) yielded 72 matched pairs with greatly improved covariate balance (standardized mean difference in Calculus II scores: 0.08, from 0.56). The OBE advantage remained significant in the matched sample (*d* = 0.36), though attenuated relative to the unadjusted estimate (*d* = 0.62), as expected when baseline differences are removed (Austin, 2011; Austin and Stuart, 2015).

##### 5.4.2.3 Convergent evidence across three analytical approaches

As reported in Section 4, the OBE effect was consistent across analytical approaches: unadjusted comparison (*d* = 0.62), ANCOVA-adjusted (*B* = 6.89, see Table 6), and propensity-matched comparison (*d* = 0.36). The expected attenuation from unadjusted to matched estimates is appropriate and substantively meaningful, as it isolates the adjusted instructional association from baseline ability differences. Additionally, a random-intercept HLM (Table 7) confirmed that the OBE advantage persisted after accounting for class-level clustering (ICC = 0.106; β = 2.324, *p* = 0.041). Taken together, this convergence of evidence across methods materially strengthens causal inference beyond what any single analytic approach could provide.

#### 5.4.3 Residual limitations and future directions

Despite the above safeguards, several limitations remain. First, the study is quasi-experimental rather than a randomized controlled trial, and unmeasured confounders that differ systematically across colleges and majors (e.g., departmental culture, peer norms, career aspirations) cannot be entirely excluded. The baseline difference in prior achievement, while plausibly attributable to chance imbalance, required statistical correction; residual confounding after adjustment cannot be fully ruled out.

Second, although we accounted for class-level clustering via random-intercept HLM, class sections may still differ in unmeasured contextual factors (e.g., peer norms, local learning climate). Future studies should include multiple instructors and institutions to disentangle instructional modality from broader contextual influences.

Third, the mediators (engagement and self-efficacy) and the perceived learning outcomeswere all measured at the same time point near course completion. Therefore, the mediationanalyses should be understood as identifying statistically meaningful indirect associationsrather than establishing causal mechanisms or temporal processes (Maxwell and Cole, 2007; Cole and Maxwell, 2003). Longitudinal designs with repeated measures are needed to test whether changes inengagement and self-efficacy temporally precede changes in learning outcomes.

Fourth, the HTMT ratios for several construct pairs approached the conservative 0.85 threshold, suggesting potential construct proximity. This issue may be addressed in future research by developing more differentiated measures (e.g., through think-aloud protocols and cognitive interviewing) and by incorporating higher-order factor models or behavioral indicators (e.g., learning analytics, observed participation) to provide a more robust test of discriminant validity (Willis, 2004; Henrie et al., 2015).

Fifth, the self-reported survey measures of engagement, self-efficacy, and perceived learning outcomes are susceptible to common method bias and social desirability effects (Podsakoff et al., 2012). The elevated correlations between certain construct pairs (e.g., ENG–SAA, SE–CDT) may partially reflect shared method variance. Although the inclusion of objective final examination scores provides external criterion information, future research should incorporate more behaviorally anchored engagement indicators (e.g., learning analytics, observed participation) and performance-based assessments of application ability and cross-disciplinary thinking (Henrie et al., 2015).

Sixth, the study was conducted at a single Chinese university with culturally specific pedagogical elements (Chinese proverbs, the “Detective Di Renjie” problem-solving framework). Cross-institutional and cross-cultural replications are needed to establish external validity. While the underlying principles of OBE and the proposed dual-pathway model may generalize, the extent of generalization and the role of cultural adaptation warrant empirical verification across contexts (Hofstede, 2011). Moreover, this study focused on liberal arts and social science students; the effectiveness of the OBE reform for STEM students, who may have different prior mathematical preparation and learning orientations, remains an open question.

Seventh, the study measured outcomes at course completion, capturing short-term associations but not long-term retention or transfer. Longitudinal research is needed to examine whether OBE-related advantages persist over time and transfer to subsequent courses and professional contexts. The durability of self-efficacy gains and cross-disciplinary thinking development is particularly important to assess (Gegenfurtner, 2013).

Future research should address these limitations through: (1) cluster-randomized designs at the section or instructor level; (2) multi-institutional replications across diverse cultural and disciplinary contexts; (3) longitudinal designs with repeated measures of engagement, self-efficacy, and learning outcomes; (4) incorporation of behaviorally anchored engagement indicators and performance-based assessments; and (5) moderator analyses examining whether OBE effects vary by student characteristics (prior achievement, major, gender).

### 5.5 External validity and generalizability

The findings of this study are bound by the specific institutional, disciplinary, and cultural context in which they were generated. We emphasize that the following discussion of contextual factors is intended to delineate boundary conditions for generalization rather than to imply that our findings automatically extend to other settings. Researchers and practitioners should exercise caution in applying these results to contexts that differ substantially from ours.

#### 5.5.1 Contextual boundaries

This study was conducted at a private comprehensive university in Eastern China, with students from liberal arts and social science majors. Several contextual factors may influence generalizability:

**Institutional context**: Private universities in China typically have smaller class sizes and more flexible curriculum design compared to public universities (Hofstede, 2011). This structural difference may enhance the feasibility of student-centered OBE strategies (e.g., small-group collaborative learning, individualized feedback) that are more challenging to implement in larger public university classes with standardized curricula. Replication in public institutions with larger classes and more standardized curricula is warranted to test the robustness of our findings.

**Disciplinary context**: The effectiveness of OBE reform may vary across mathematical subjects. Probability and statistics naturally lend themselves to real-world applications, which may enhance OBE effectiveness by providing tangible learning outcomes for students (Dinov et al., 2008). Future research should examine whether similar patterns emerge in more abstract mathematical courses (e.g., real analysis, abstract algebra) where authentic outcome demonstration is more challenging, as the dual-pathway model (engagement/self-efficacy) may operate differently for non-applied mathematical content.

**Student population**: Liberal arts students often have lower prior mathematics preparation (Li and Li, 2024), which may make them more responsive to OBE's scaffolded approach (e.g., visual/tabular probability calculations, cultural integration) designed to reduce cognitive load. STEM students, with stronger mathematical foundations, may require more advanced interdisciplinary OBE applications (e.g., machine learning integration) to achieve comparable effects. Testing the model with STEM students would help determine whether the dual-pathway mechanism generalizes across student populations with different mathematical backgrounds.

**Temporal ordering and mediation interpretation**:A fundamental limitation of the present mediation analysis is that the mediators (engagement and self-efficacy) and the endogenous outcomes (KM, SAA, CDT) were measured at the same time point (end-of-semester survey). This cross-sectional measurement design does not satisfy the temporal ordering requirement for causal mediation analysis, as articulated by Maxwell and Cole (2007); Cole and Maxwell (2003). Without evidence that changes in engagement and self-efficacy temporally preceded changes in perceived learning outcomes, the observed indirect effects could reflect: (a) the hypothesized causal sequence (OBE → ENG/SE → outcomes), (b) reverse causation (positive learning experiences → higher engagement/self-efficacy reports), or (c) a common unmeasured cause that simultaneously influences all constructs. We therefore interpret the mediation results as identifying *statistically consistent indirect associations* that align with the theoretical model, while emphasizing that causal mediation remains an empirical question for future longitudinal research.

#### 5.5.2 Cultural specificity of pedagogical elements

A distinctive feature of the OBE reform in this study is the integration of culturally-specific elements, including Chinese proverbs and idioms (e.g., “Sai Weng's Lost Horse” for conditional probability), the “Detective Di Renjie” problem-solving framework, and connections to national development strategies through curriculum ideology and politics.

These elements were designed to enhance engagement and relevance for Chinese students within the New Liberal Arts context. Their effectiveness in other cultural settings is an empirical question. However, the *principle* of cultural integration, which involves connecting abstract mathematical concepts to culturally meaningful contexts, is likely generalizable, even if specific examples require adaptation (Nasir et al., 2008; Gay, 2018; Franco et al., 2024).

#### 5.5.3 Transferability of core findings

Despite contextual specificity, several core findings are likely transferable:

1. The dual-pathway model (ENG → KM/SAA; SE → CDT) reflects general psychological mechanisms (social cognitive theory, engagement theory) that should operate across contexts, regardless of institutional or disciplinary differences.
2. The importance of aligning learning outcomes, instructional activities, and assessment (constructive alignment) is a universal pedagogical principle (Biggs, 2003), and our findings reinforce its value in mathematics education for diverse student populations.
3. The finding that higher-order outcomes (CDT) depend critically on self-efficacy has implications for any educational context seeking to promote transfer and integration, particularly in interdisciplinary education reform efforts.

Future research should test these mechanisms across diverse cultural and institutional settings to establish boundary conditions and identify necessary adaptations (e.g., cultural recontextualization of pedagogical elements, scaling of scaffolded instruction for large classes).

#### 5.5.4 Summary of generalizability constraints

To summarize, the external validity of this study is constrained by at least five factors: (1) single institutional context (a private comprehensive university in Eastern China); (2) specific student population (liberal arts and social science majors); (3) single disciplinary focus (probability and statistics); (4) culturally specific pedagogical elements that may not transfer directly to other cultural contexts; and (5) single-semester timeframe that precludes assessment of long-term effects. While the underlying theoretical mechanisms (engagement and self-efficacy pathways) are grounded in well-established psychological theories with broad applicability, the *magnitude* and *pattern* of effects observed in this study may not generalize beyond the boundary conditions specified above. Multi-site, cross-cultural, and longitudinal replications are essential to establish the robustness and generalizability of the proposed dual-pathway model.

## 6 Conclusion

This study provides empirical evidence consistent with the proposition that Outcome-Based Education reform is positively associated with student learning outcomes in probability and statistics courses within the New Liberal Arts context. Through statistical indirect pathways involving engagement and self-efficacy, OBE is associated with knowledge mastery, statistical application ability, and cross-disciplinary thinking, though these associations should be interpreted cautiously given the cross-sectional and quasi-experimental design.

The positive associations observed for culturally-integrated, student-centered approaches in this study offers both theoretical insights into the mechanisms of educational effectiveness and practical guidance for curriculum design and pedagogical innovation. The finding that self-efficacy is particularly critical for cross-disciplinary thinking, accounting for over 74.0% of the total effect, highlights the importance of psychological empowerment in achieving the integrative competencies emphasized by New Liberal Arts education.

As higher education continues to evolve in response to societal demands for interdisciplinary competencies and data literacy, OBE provides a promising framework for transforming mathematics education. The success of culturally-integrated, student-centered approaches demonstrated in this study suggests that effective pedagogy must attend not only to what students learn but also to how they experience the learning process.

Future research should extend these findings through longitudinal designs, cross-cultural replications, and integration of objective outcome measures. Ultimately, the goal is to develop evidence-based pedagogical approaches that prepare students not merely to master mathematical techniques but to apply quantitative reasoning across the diverse challenges they will encounter in their academic and professional lives.

## Data Availability

Publicly available datasets were analyzed in this study. This data can be found here: Link: https://pan.baidu.com/s/1hO8AlKZxCboAemSpGeBxUQ?pwd=aphg Extraction Code: aphg.
