# Supplementary material for: Outcome-based education and student learning in probability and statistics: the mediating roles of engagement and self-efficacy in a new liberal arts context
Source: Front Psychol. 2026 May 5;17:1817477. doi: 10.3389/fpsyg.2026.1817477 (PMC13183824; doi:10.3389/fpsyg.2026.1817477)
Supplement: Supplementary file 1 [file Table_1.pdf]

Outcome-Based Education and Student Learning in Probability and Statistics: The Mediating Roles of Engagement and Self-Efficacy in a New Liberal Arts Context

Table of Contents(Appendix)

A.1 Questionnaire 1

A.2 Course Design Framework 2

Appendix 1: Questionnaire

Table 1: Questionnaire Items, Content, and Response Counts Across Various Learning Dimensions (Including Course Model, Knowledge Mastery, Engagement, Self-Efficacy, Statistical Application Ability, and Cross-Disciplinary Thinking)

Item Number	Item Content	Source
Course Model (CM)		
	Traditional Teaching	
	OBE-Reformed Course Teaching	
Knowledge Mastery (KM)		(Biggs, 2003)
KM1	I can easily understand the basic concepts of probability and statistics taught in this course.	
KM2	Using common sayings and proverbs helps me master probability concepts more effectively.	
KM3	Contemporary cases (such as infectious disease models) make the course content more relevant and easier to understand.	
KM4	Simplified methods (such as tabular and graphical methods) help me learn complex probability concepts.	
KM5	I am confident in conditional probability and its applications.	
KM6	The way the course content is structured facilitates my learning.	
Engagement (ENG)		(Fredricks et al., 2004)
ENG1	I find the course activities engaging and enjoyable.	
ENG2	Using Detective Di Renjie's problem-solving approach makes the questions more interesting.	
ENG3	I actively participate in online discussions and interactive activities.	
ENG4	The course content (such as common sayings and contemporary cases) keeps me interested in learning.	
ENG5	I like activities like "The Course Ideology and Politics from Students' Perspectives".	
ENG6	The course creates a fun and interactive learning environment.	
Self-Efficacy (SE)		(Schwarzer and Jerusalem, 1995)
SE1	After this course, I feel confident in my ability to solve complex probability problems.	
SE2	The course activities have enhanced my confidence in problem-solving skills.	
SE3	I feel capable of applying probability concepts to real-world problems.	
SE4	The course ideology and politics section has boosted my confidence in my learning ability.	
SE5	I believe I can effectively handle challenging statistical problems.	
SE6	The course has enhanced my overall confidence in my learning ability.	
Statistical Application Ability (SAA)		(Heppner and Petersen, 1982)
SAA1	I can apply probability concepts to solve real-world problems, such as simulating the spread of infectious diseases.	
SAA2	Course activities (such as solving mixed distribution problems) have improved my ability to apply statistical knowledge.	
SAA3	I can use probability models to analyze complex systems and their interdependencies.	
SAA4	The course has helped me develop skills to simplify complex problems using tabular and graphical methods.	
SAA5	I am confident in applying probability and statistics to real-world scenarios.	
SAA6	The course provides opportunities to meaningfully practice applying statistical knowledge.	
Cross-Disciplinary Thinking (CDT)		(Spelt et al., 2009)
CDT1	The course has helped me see the connections between probability, ideological education (such as national strategies), and personal development.	
CDT2	I can integrate probability knowledge with my major or professional field.	
CDT3	Common sayings and proverbs have helped me understand the impact of probability in a broader society.	
CDT4	The course activities encourage me to think about probability from a cross-disciplinary perspective.	
CDT5	I can apply probability concepts to analyze problems in my professional field.	
CDT6	The course has enhanced my ability to connect mathematical knowledge with real-world social issues.	

Appendix 2: Course Design Framework

Reference: Adapted from trust repair research (Schweitzer et al., 2006; Tomlinson & Mayer, 2009)

Table 2: Course Design Framework for "Probability Theory and Mathematical Statistics" under OBE Concept in the Context of New Liberal Arts

**Item**	**System Structure**	**Research Objective Classification**	**Research Content/Implementation Knowledge Points**
Reform and Exploration of "Probability Theory and Mathematical Statistics" Course under OBE Concept in the Context of New Liberal Arts	Course Content System	Proverbs and Aphorisms	Trust decay and reconstruction from the fable "Cried Wolf" / Bayesian formula<br>"Three cobblers with their wits combined equal Zhuge Liang" / Compound events<br>"Strike first to gain advantage, delay to suffer disadvantage" / Event independence
		Curriculum Ideology and Politics	Conditional probability and compound events-Understanding China's Three-Step Development Strategy<br>Principle of "Guessing answers in exams won't lead to passing" / Binomial distribution<br>"Principle of small probability events"-Capacity for value judgment, anti-fraud awareness
		Contemporary and Life-Related Cases	Epidemic transmission probability models<br>Principle of nucleic acid pool testing / Mathematical expectation<br>Analyzing reasons for grade differences between two classes in simultaneous courses / Hypothesis testing
	Course Competency System	Reverse Thinking	Reverse calculation of "at least one hit"
		Logical Thinking	Logical deduction of "air combat scenarios"
		Exploratory Ability	"Three Doors Problem" (Let's Make a Deal)<br>Programming calculations for binomial, Poisson, and normal distributions
		Modeling Ability	Time window modeling for "meeting problems"
		Transformation Ability	Binomial distribution table transformation<br>Density function integration region transformation
		Systems Thinking Ability	Distribution and expectation analysis of random variable functions
	Course Methodology System	Problem-Driven Learning	Detective-style "case within a case" analysis
		Complex Problem Simplification	"Four-ball drawing" problem / Tabular/graphical solutions
		Scientific Research Methods	Multidimensional random vector analysis framework<br>Hypothesis testing and regression analysis
		Exercise Training	Task-based exercise training system
		Post-Class Summary	Mind mapping for chapter knowledge
	Course Evaluation System	Essays and Reading Reports	Essays on "Approaching Curriculum Ideology and Politics"<br>Reports on "Curriculum Ideology and Politics from Students' Perspectives"<br>Perceptions of philosophical wisdom in proverbs from probability theory perspective
		Online Discussions	Rain Classroom online Q&A discussions
		Classroom Effectiveness Evaluation	Multi-dimensional teaching quality evaluation
		Class Participation and Assignments	Class participation and assignment training components
		Assessment Methods	Comprehensive final exam evaluation

References

Biggs, J. (2003). Aligning teaching and assessing to course objectives. *Teaching and learning in higher education: New trends and innovations*, 2(4):13–17.

Fredricks, J. A., Blumenfeld, P. C., and Paris, A. H. (2004). School engagement: Potential of the concept, state of the evidence. *Review of educational research*, 74(1):59–109.

Heppner, P. P. and Petersen, C. H. (1982). The development and implications of a personal problem-solving inventory. *Journal of counseling psychology*, 29(1):66.

Schwarzer, R. and Jerusalem, M. (1995). Generalized self-efficacy scale. *J. Weinman, S. Wright, & M. Johnston, Measures in health psychology: A user's portfolio. Causal and control beliefs*, 35(37):82–003.

Spelt, E. J., Biemans, H. J., Tobi, H., Luning, P. A., and Mulder, M. (2009). Teaching and

learning in interdisciplinary higher education: A systematic review. *Educational Psychology Review*, 21:365–378.
